# Titanium Dioxide Nanoparticles: Prospects and Applications in Medicine

**DOI:** 10.3390/nano10020387

**Published:** 2020-02-23

**Authors:** Daniel Ziental, Beata Czarczynska-Goslinska, Dariusz T. Mlynarczyk, Arleta Glowacka-Sobotta, Beata Stanisz, Tomasz Goslinski, Lukasz Sobotta

**Affiliations:** 1 Department of Inorganic and Analytical Chemistry, Poznan University of Medical Sciences, Grunwaldzka 6, 60-780 Poznan, Poland; dziental@ump.edu.pl; 2 Department of Pharmaceutical Technology, Poznan University of Medical Sciences, Grunwaldzka 6, 60-780 Poznan, Poland; bgoslinska@ump.edu.pl; 3 Department of Chemical Technology of Drugs, Poznan University of Medical Sciences, Grunwaldzka 6, 60-780 Poznan, Poland; mlynarczykd@ump.edu.pl; 4 Department and Clinic of Maxillofacial Orthopedics and Orthodontics, Poznan University of Medical Sciences, Bukowska 70, 60-812 Poznan, Poland; aglow@ump.edu.pl; 5 Department of Pharmaceutical Chemistry, Poznan University of Medical Sciences, Grunwaldzka 6, 60-780 Poznan, Poland; bstanisz@ump.edu.pl

**Keywords:** composites, nanoparticles, photodynamic therapy, photosensitizer, titanium dioxide

## Abstract

Metallic and metal oxide nanoparticles (NPs), including titanium dioxide NPs, among polymeric NPs, liposomes, micelles, quantum dots, dendrimers, or fullerenes, are becoming more and more important due to their potential use in novel medical therapies. Titanium dioxide (titanium(IV) oxide, titania, TiO_2_) is an inorganic compound that owes its recent rise in scientific interest to photoactivity. After the illumination in aqueous media with UV light, TiO_2_ produces an array of reactive oxygen species (ROS). The capability to produce ROS and thus induce cell death has found application in the photodynamic therapy (PDT) for the treatment of a wide range of maladies, from psoriasis to cancer. Titanium dioxide NPs were studied as photosensitizing agents in the treatment of malignant tumors as well as in photodynamic inactivation of antibiotic-resistant bacteria. Both TiO_2_ NPs themselves, as well as their composites and combinations with other molecules or biomolecules, can be successfully used as photosensitizers in PDT. Moreover, various organic compounds can be grafted on TiO_2_ nanoparticles, leading to hybrid materials. These nanostructures can reveal increased light absorption, allowing their further use in targeted therapy in medicine. In order to improve efficient anticancer and antimicrobial therapies, many approaches utilizing titanium dioxide were tested. Results of selected studies presenting the scope of potential uses are discussed in this review.

## 1. Introduction

The intensive development of photodynamic therapy (PDT) in recent years has involved the search for new photosensitizers and specific carriers for their delivery. Among many promising approaches to be noted for photodynamic research, those in which dyes and nanoparticles (NPs) were combined led to an increase in the selectivity of the photosensitizer (PS) and/or efficacy of the therapy.

At the very beginning, it should be explained that NPs constitute a particular type of particles of the size between 1 and 100 nm (with the surrounding interfacial layer) [1]. The exact definition given in ISO technical specification 80004 states that NPs are "*nano-objects with all three external dimensions in the nanoscale, whose longest and shortest axes do not differ significantly*". It should be noted that in the broad sense, NPs also include polymeric NPs, liposomes (multilayer), lipid micelles (unilayer), quantum dots, dendrimers, fullerenes, cubosomes, niosomes, and metallic NPs. Particular attention should be paid to the last mentioned, but exceptional category containing metallic and metal oxide NPs, for example ZnO, Au, Fe_2_O_3_, TiO_2_. Several studies have indicated that the application of NPs in medicine can significantly improve the effectiveness of many existing therapies. Linking drugs with NPs can enhance their selective accumulation in diseased tissues as well as penetration abilities through cell membranes. Increasing the selectivity of drugs is a great challenge for modern medicine. This goal can be achieved by research focused on therapeutic systems with increased selectivity and reduced toxicity, accompanied by higher therapeutic efficiency [2,3].

**Figure 1:**
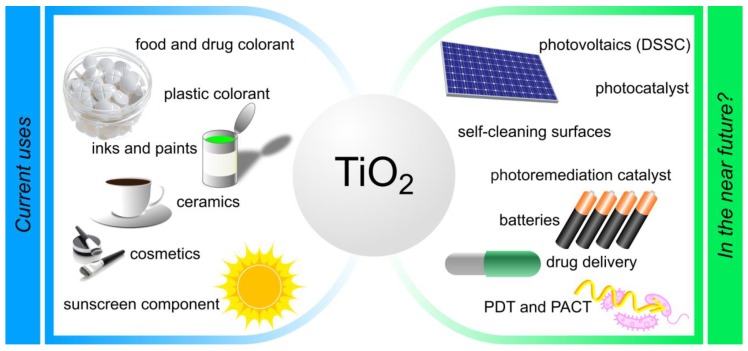
Current applications and potential future use of TiO_2_. PDT, photodynamic therapy; PACT, antimicrobial photodynamic therapy; DSSC, dye-sensitized solar cell.

In the current review, studies focusing on titanium dioxide (titanium(IV) oxide, titania, TiO_2_) nanoparticles, which belong to the category of metallic NPs are reviewed. Notably, the evaluation of current TiO_2_ functionalization methods accompanied by the biological and medical effects of these NPs were the driving force for this work. Mass production of TiO_2_ began in the early twentieth century as a non-toxic substitute for a white dye for paints. Nowadays, the annual production of TiO_2_ exceeds four million tons per year, and this molecule has found numerous applications in everyday products (Figure 1)—as an excipient in the pharmaceutical industry, for sun cream production in the cosmetics industry, as a colorant in white plastics, and as a relatively cheap and nontoxic food pigment approved by the relevant European Union authorities for the safety of food additives [4]. Research on the possible applications of TiO_2_ NPs dates back to 1985 when one of the first works on the subject of photocatalytic disinfection was published [5]. Since that time, the use of TiO_2_ NPs in photodynamic therapy studies has been constantly increasing. It concerns TiO_2_ NPs’ applications as photosensitizing agents in the treatment of cancer as well as in photodynamic inactivation of antibiotic-resistant bacteria. Both TiO_2_ NPs themselves, as well as their composites, combinations, or hybrids with other molecules, were successfully tested as photosensitizers in photodynamic therapy. Titanium(IV) oxide NPs were applied *inter alia* in the synthesis of bioconjugates with cell-specific monoclonal antibodies for the treatment of malignant tumors or the preparation of black TiO_2_ NPs for antimicrobial therapy of antibiotic-resistant bacteria [6,7].

## 2. Pharmacokinetics, Biodistribution, and Biological Fate of Titanium Dioxide

At present, relatively few publications have addressed issues related to TiO_2_ NPs’ pharmacokinetic characteristics. Also, some of the literature reports are contradictory or ambiguous. The pharmacokinetics of metal NPs, including TiO_2_, depends on many factors, including particle type, surface charge, surface coating, size, dose, and exposure route [8,9].

Titanium dioxide can generally enter the body in three ways: orally, transdermally, or by injection. Research and discussion on the bioavailability of TiO_2_ from the gastrointestinal tract are currently underway. There are many indications that titania does not penetrate the gastrointestinal tract at all or to a minimal extent. Animal studies have shown that 24 h after oral administration of TiO_2_ NPs at a dose of 100 mg per kilogram of body weight, no significant increase in NP concentration in any of the tested tissues was detected [10]. Analogous studies using even higher doses of TiO_2_ gave similar results confirming that orally administered TiO_2_ does not penetrate the gastrointestinal tract and that penetration is medically insignificant [11]. However, studies using the physiologically-based pharmacokinetic modeling technique have indicated that high concentrations of TiO_2_ NPs could lead to their agglomeration and thus increase their uptake by macrophages. As indicated by Bachler et al. [12], the biodistribution of TiO_2_ NPs can proceed via two kinetic processes utilizing their ability to penetrate through the blood vessels to the organs and by phagocytosis of NPs by the mononuclear phagocyte system. However, it should be emphasized that the pharmacokinetics of NPs after intravenous administration is different [12]. As in such case, the bioavailability of NPs is complete, their distribution in the body should be carefully considered. In a study performed by Fabian et al. [13], rats were administered intravenously with 5 mg TiO_2_ NPs per kg of body weight and then observed for 28 days. The animals were healthy and behaved normally throughout the test period. Histopathological study revealed that TiO_2_ did not accumulate at detectable levels in blood cells, plasma, brain, or lymph nodes. However, titania levels were the highest in the liver, while lower, but still elevated concentrations were observed in the spleen, lungs, and kidneys [13].

An essential observation concerning TiO_2_ NPs excretion by the kidneys in rats was made by Geraets et al. [14]. They noticed that TiO_2_ is eliminated from the body slowly, which indicates its potential tissue accumulation. This issue is not severe considering PDT because the photosensitizer is administered only once or several times during the photodynamic therapy [14]. Besides, the study performed by Xie et al. [15] on rats showed that the TiO_2_ NPs level in urine was higher than in feces, indicating renal excretion as the primary route of TiO_2_ NP elimination [15].

❖ **To sum up**

○ The pharmacokinetics of TiO_2_ NPs depends on many factors, including particle type, surface charge, surface coating, size, dose, and exposure route.
○ Titania does not penetrate the gastrointestinal tract at all or to a minimal extent.
○ Histopathological study indicates that after intravenous administration TiO_2_ NPs accumulate mainly in the liver, and to some extent in the spleen, lungs and kidneys.
○ Renal excretion is the primary route of TiO_2_ NPs elimination.
○ The pharmacokinetics and bioavailability of TiO_2_ NPs require further and intensive research.

## 3. Toxicity and Biocompatibility—*In Vitro* and *In Vivo* Evaluation of the Toxicity of Titanium Dioxide

The wide application of titanium dioxide is related to its low toxicity. Many studies with TiO_2_ of different nanoparticle and microparticle sizes and crystal forms were performed to assess skin, lung, immune system, and hematotoxicity. Although titania is a quite common ingredient of many cosmetic formulations, especially sunscreens, powders, and eyeshadows, it seems that its size and crystal forms (anatase and rutile) influence the safety of its usage.

The *in vitro* and *in vivo* studies concerning the skin-related toxicity of TiO_2_ NPs raised two issues, namely skin toxicity itself and systemic toxicity associated with skin permeation. Wu et al. studied the toxicity and penetration of TiO_2_ NPs in hairless mice and porcine skin after subchronic dermal exposure [16]. According to the presented findings, the researchers concluded that the nanosized TiO_2_ might pose a health risk to humans after chronic dermal exposure over a relatively long period, mainly due to deeper tissue distribution. In another study, Crosera et al. studied both TiO_2_ NPs’ penetration on Franz cells for 24 h using intact and needle-abraded human skin as well as evaluated cytotoxicity on HaCaT keratinocytes. The study demonstrated that the presence of TiO_2_ NPs was limited to the epidermal layer, whereas in the dermal layer, their concentration was below the limit of detection. A slight cytotoxic effect on human HaCaT keratinocytes was noted suggesting the potential TiO_2_ NPs related risk only after long-term exposure [17]. In a related study, Yin et al. analyzed the phototoxicity of TiO_2_ NPs with different molecular sizes and crystal forms (anatase and rutile) in HaCaT human skin keratinocytes [18]. The outcomes indicated that TiO_2_ NPs are phototoxic to human skin keratinocytes as the result of emergence of reactive oxygen species (ROS) generated during UVA irradiation. It is important to note that the rutile form of nano-TiO_2_ revealed less phototoxicity than anatase [18].

The potential risk related to TiO_2_ inhalation exposure has been the subject of many studies. The toxicity study revealed mainly some adverse effects related to titania, also in experiments that could indicate significant “overload”. A study conducted by Lee et al. can be given as an example [19]. It was found that long-term inhalation exposure of rats to bulk TiO_2_ dust at high concentration (up to 250 mg/m^3^ for 2 years, 6 h/day for 5 days/week) caused bronchioloalveolar adenomas and cystic keratinizing squamous cell carcinomas. Due to the specific nature of the relevant pre-malignant tumors, atypical for human lung cancer, and lack of tumor metastases to other organs, the researchers concluded that the observed tumors arose from the excessive dust loading in the lungs, so-called TiO_2_ “overload” [19]. A very interesting research related to the potential risk of inhalation exposure has been recently reported by Vandebriel et al. [20], who studied TiO_2_ NPs, which are also a common material applied in paints during their production or applications. Researchers studied TiO_2_ NPs in terms of their immune activity *in vitro* and *in vivo*. The first section of the study was concentrated on the *in vitro* assessment of TiO_2_ NPs on the maturation of dendritic cells, which form an important part of the lung immune system, whereas the second section was related to the research performed on their adjuvant activity *in vivo* on mice. For the study, a series of fourteen TiO_2_ NPs were chosen, differentiated in terms of crystal structures and coatings. Rutile form of TiO_2_ NPs was found to be safer than anatase NPs as *in vitro* anatase and anatase/rutile TiO_2_ NPs induced a higher expression of CD83 and CD86 and a higher production of IL-12p40, than rutile NPs. In this way, the maturation of dendritic cells was induced to a greater extent by anatase and anatase/rutile NPs than by rutile NPs. This finding is important in terms of further choice of titanium dioxide crystal structure for the applications in industry, especially in the areas, where the inhalation exposure during production or application of the product should be considered [20]. It is known that the stimulation of dendritic cell maturation can lead directly to a whole cascade of physiological phenomena, including a specific immune response, and indirectly to inflammation [21]. Continuous exposure to TiO_2_ can therefore potentially lead to an excessive immune response and chronic inflammation. Chronic inflammation is considered a harmful state, responsible for the destruction of the body’s tissues and the development of other diseases [22]. Complementary research presenting the role of inflammatory processes was conducted by Madhubala et al., who studied *in vitro* cytotoxic and immunomodulatory effects of the low concentration of TiO_2_ NPs on various human cell lines [23]. The immunomodulatory effects of TiO_2_ NPs were tested on human monocytic leukemia (THP-1) and human mast (HMC-1) cell lines in a dose-dependent manner. The viability of THP-1 cells treated with titania NPs was significantly reduced at higher doses as indicated in MTT (3-(4,5-dimethylthiazol-2-yl)-2,5-diphenyltetrazolium bromide) assay. What is interesting, the secretion of cytokines (IL-6 and IL-10) by human cell lines was significantly correlated with the concentration of TiO_2_ NPs. Titania NPs at lower concentrations induced inflammatory responses in studied cells through cytokine secretion [23].

A very interesting problem, vitally important in this review, is related to the issue of whether titanium dioxide toxicity can be modified by the combination of these NPs with porphyrinoids. This question cannot be unambiguously answered because further research is needed in the coming years. Nevertheless, some studies, like that performed by Rehman et al. can be considered at a very initial stage as promising [24]. The authors assessed the importance of TiO_2_ nanowhiskers in combination with 5,10,15,20-tetra(4-sulfonatophenyl)porphyrin (TSPP) *in vivo* on rats. What is essential, TSPP applied in the above-discussed experiment is considered as a photosensitizer, which is not free of side effects. In the study, different concentrations of either TSPP, TiO_2_, or hybrid TSPP-TiO_2_ material were used. Toxic properties were assessed based on fluorescent microscopy, complete blood cells count, and serum enzymes, which allowed the evaluation of the effect of excretory and circulatory systems. The TSPP and TSPP-TiO_2_-treated rat groups were also illuminated with visible light (500–550 nm). Based on all the above-mentioned tests, it turned out that the combination of TiO_2_ NPs with the porphyrin significantly reduced TSPP toxicity, especially at high concentrations. It was clearly demonstrated that TSPP (0.1 mM) combined with TiO_2_ nanowhiskers (0.6 mM) was safer than TSPP (0.1 mM) alone. According to the MTT assay, TSPP combined with TiO_2_ nanowhiskers revealed minimized cytotoxic effects on the normal cells in terms of increased viability. This protection of the TiO_2_ nanowhiskers was attributed to their porous nature allowing a slow release of the adsorbed TSPP into the surrounding environment, thus helping in lowering adverse effects without compromising the theranostic properties [24].

❖ **To sum up**

○ The toxicity of titanium dioxide is low. Various studies consider this material as safe or unsafe, depending on the size and crystal form, which strongly determines TiO_2_ NPs’ potential toxicity.
○ The *in vitro* and *in vivo* studies concerning the skin-related toxicity of TiO_2_ NPs raise both skin toxicity itself and skin permeation related systemic toxicity. The potential TiO_2_ NPs related risk on skin after long-term exposure cannot be neglected.
○ The harmful effects of TiO_2_ inhalation exposure are associated with the so-called TiO_2_ “overload”, which is rare in everyday life.
○ Some immunomodulation effects related to the stimulation of dendritic cell maturation by TiO_2_ presented in recent studies cannot be omitted.
○ It seems that TiO_2_ toxicity can be modified by combining it with photosensitizers.

## 4. Design of Titanium Dioxide Nanoparticles—Synthesis and Stabilization Procedures, Physicochemical Properties, and Characterization

Titanium dioxide is a metal oxide that naturally occurs in nature [25]. Named after two of the most abundant minerals, the two most common tetragonal crystallographic polymorphs of TiO_2_ take their name from—anatase and rutile. The third, rarer orthorhombic crystal structure, belongs to brookite. Titanium(IV) oxide is mostly produced by the purification of rutile mineral, or by subjecting ilmenite (FeTiO_3_) to either so-called chloride or sulfate processes, which both finally yield pure titania. When the thermal treatment is applied, the amorphous TiO_2_ may be transformed into anatase or brookite in a process called calcination, which takes place at around 400 °C [25,26,27]. However, these polymorphs, when heated to temperatures above 600 °C, are converted to rutile.

**Figure 2:**
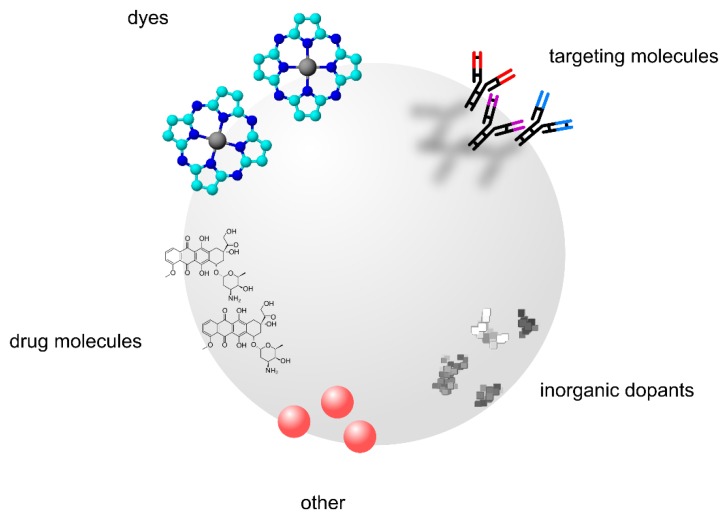
The spectrum of possible TiO_2_ nanoparticles modification for medicinal purposes.

The synthetic methods leading to TiO_2_ in general as well as to titania NPs include a series of techniques, with sol-gel synthesis and hydrothermal methods being the most frequently applied [26,27], green chemistry and microwave methods are on the rise [28,29]. By careful design and modification of the process parameters (i.e., substrates used, ratio of solvents, temperature, process time), it is possible to obtain the desired materials with varying specific physicochemical properties, such as surface area, NP morphology and form, NP size and uniformity in size distribution, crystallinity and crystal phase, photoactivity, and many others. These properties can be additionally modified during the synthesis of NPs by the addition of various surfactants or dopants or by post-synthetic modifications, such as doping, surface functionalization, or binding with organic molecules (Figure 2) [25,27].

**Figure 3:**
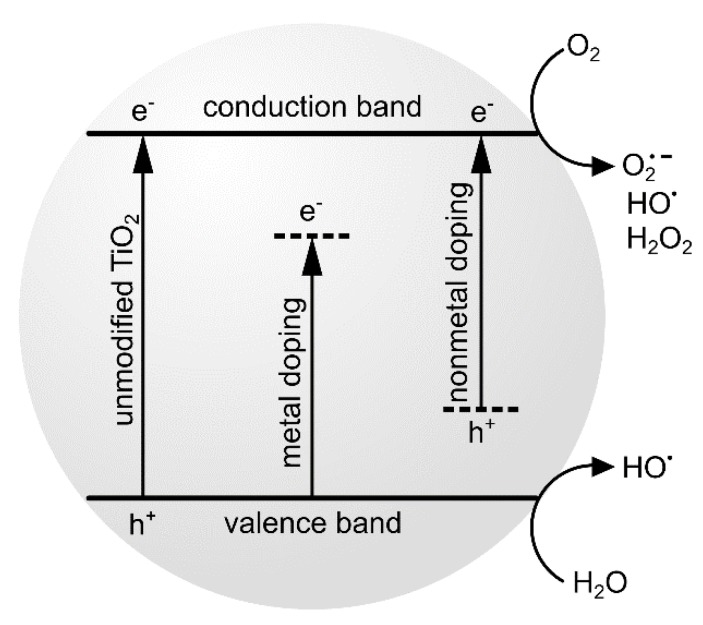
Simplified mechanism of reactive oxygen species generation by TiO_2_ (based on [37]).

Despite the promising properties of this material for photodynamic therapy, research is still underway to modify the NPs’ surface in order to increase the efficiency of ROS generation and to improve the physicochemical properties, including the visible light absorption. Surface-modified TiO_2_ NPs with photosensitizing properties create a potential for PDT [30]. Effective photosensitization with the use of a wide band-gap semiconductor TiO_2_ has appeared in many studies aiming to extend its spectral response. Therefore, titanium dioxide can be doped with various metal ions and non-metal dopants [31,32] or combined with various dyes [33,34,35]. Surface complexes acting as TiO_2_ photosensitizers usually include transition metal ion with inorganic or organic ligands. The organic ligands are coordinatively bound to the central ion and covalently linked to the titanium dioxide surface. Inorganic ligands, such as CN^–^, F^–^, PO_4_^3^^–^ can also link surface titanium with metal centers. The photosensitization is the effect either of the photoinduced electron injection from the surface of the complex to the conduction band of the semiconducting support or of a hole injection to the valence band. Photoinduced charge injection can base on direct or indirect photosensitization processes. In some cases, the complexes formed at titanium dioxide surface can be obtained upon chemisorption due to the presence of anchoring groups in the structure of organic molecules [30]. The relevant titanium dioxide is a semiconductor-based material with an energy gap of 3.23 eV for anatase and 3.06 eV for rutile polymorph [2,6]. If the molecule absorbs a photon with energy higher or equal to that value, it passes to an excited state and can produce negative electrons in the conduction band, leaving positively charged holes in the valence band. Free electrons may attack surrounding oxygen and water molecules to form ROS, including superoxide (O_2_^•-^), hydrogen peroxide (H_2_O_2_), and hydroxyl radical (^•^OH) (Figure 3) [36]. These forms of oxygen are highly unstable in biological systems and react with the cell components causing apoptotic or necrotic cell death. It has also been proven that TiO_2_ NPs inhibit efflux-mediated multidrug resistance [36].

Due to the nature of titania NPs when dispersed in aqueous solutions, in most cases, they tend to form agglomerates [38,39]. These forms have a decreased surface area and thus reveal also lower photoactivity. In addition to the biological activity of TiO_2_ NPs, sedimentation may lower their concentration and interfere with the reproducibility of the results, as well as prevent the steady dosage. Therefore, stable formulations of NPs functionalized on their surface to prevent or eliminate this unwelcome property were developed. For example, the modifications of NPs rely on applying a charge for electrostatic repulsion or adsorption of stabilizers that provide a steric hindrance [40].

The TiO_2_ NPs and their aggregates can be analyzed using microscopic methods as well as size distribution techniques, such as light scattering, particle tracking analysis, or others. The techniques indispensable for the characterization of TiO_2_ nanomaterials are X-ray powder diffraction that allows studying the crystalline phase of titania and infrared spectroscopy that allows for analysis of the chemical groups present on the NP surface [40]. Another method, diffuse reflectance UV-Vis (UV-Vis DRS) spectroscopy is a useful tool for determining the light absorption spectrum of the functionalized materials. It provides the light range that can be applied to excite the NPs, thus allowing the study of their bandgap and assess their usefulness for phototherapy [29,39].

❖ **To sum up**

○ TiO_2_ occurs naturally in three polymorphic forms: rutile and anatase with a tetragonal structure, and rhombic brookite.
○ Synthetic TiO_2_ is obtained by sol-gel synthesis, hydrothermal methods, green chemistry, microwave methods, and others.
○ The TiO_2_ particles can be modified by the addition of various surfactants or dopants or by post-synthetic modifications, such as doping, surface functionalization, or binding with organic molecules.
○ Titania NPs, when dispersed tend to form agglomerates. The TiO_2_ NPs functionalized on their surface can form stable, non-aggregating formulations in aqueous solutions.

## 5. Photodynamic Activity of Neat TiO_2_ Nanoparticles and in Drug Delivery Systems

The introduction of neat titania NPs to photodynamic therapy is significantly limited by many issues related to tissue overheating under the influence of light, low tissue penetration by ultra-violet light, and harmful impact of UV radiation on the human body [7]. Neat TiO_2_ NPs and in combination with various molecules, antibodies, or polymers revealed interesting photocytotoxicity against cancer cells and microbes, thus unveiling potential for photodynamic therapy.

Although TiO_2_ is a potent oxygen radical generator, it can be excited in its pure form only by UV light. Lagopati et al. investigated the photo-induced bioactivity of titanium dioxide against cancer cells and the mechanism of action of TiO_2_ NPs [41]. The studies were conducted on the MCF-7 and MDA-MB-468 breast epithelial cancer cell lines. The aqueous dispersions of nanostructured titania were irradiated with UVA (wavelength 350 nm) for 20 min. The nanostructured TiO_2_ photosensitizer dispersions were prepared using the sol-gel technique. It is worth noting that in the TiO_2_ sols the presence of photocatalyst in the form of anatase NPs was confirmed. According to the results of the study, the applied modification revealed strong efficacy against the highly malignant MDA-MB-468 cells, which underwent apoptotic cell death. It is important to notice that the use of UV light alone caused only a 10% decrease in MDA-MB-468 cell viability, whereas non-irradiated TiO_2_ NPs at 16 µM concentration decreased the cell viability by 50%. Moreover, MCF-7 cell line was found to be resistant to this therapy under identical conditions. The observed apoptotic cell death was induced indirectly by the increase of caspase-3-mediated poly (ADP-ribose) polymerase (PARP) cleavage [41]. Non-modified titania NPs were also the subject of research of Wang et al., who investigated the effect of TiO_2_ NPs *in vitro* on glioblastoma multiforme cells upon 365 nm light irradiation and then *in vivo* on glioma-bearing mice [42]. On the one hand, it was found that the performed UV-PDT protocol resulted in higher mice survivability along with tumor growth suppression. On the other hand, despite the statistically significant effects, some critical drawbacks of UV PDT protocol with neat titania NPs were found, mostly related to the limited penetration of UV light through tissues.

It seems that an increase of the therapeutic efficiency and a reduction of drug side effects can be achieved using modern medical and pharmaceutical approaches, including the so-called smart drug delivery or targeted drug delivery systems. Such an approach improving the selectivity of NPs was proposed by Xu et al., who applied TiO_2_ NPs conjugated with a specific monoclonal antibody against the carcinoembryonic antigen of human metastatic colon adenocarcinoma (LoVo) cancer cells [6]. The obtained combination improved hybrid NPs distribution and increased the accumulation of the drug in the pathological tissues. Additionally, they used electroporation – a technique that induces the formation of micropores in biological membranes, thus increasing the membrane permeability. In these experiments, the application of a novel approach significantly enhanced the internalization of the materials, which resulted in a 100% decrease in viable LoVo cells after irradiation with ultraviolet 365 nm light, as compared to the death of 44% of the cell population after irradiation alone. Noteworthy, the particularly beneficial effects of electroporation with the electronic pulses at 500 V/cm for 100 µs of increased efficiency and specificity were noted at the very low concentration of antibody-TiO_2_ bioconjugate (even up to 3.12 μg/mL). The proposed approach can be used in the therapy of other cancer types if appropriate antibodies are matched [6]. Another way to increase the selectivity of NPs is to combine them with folic acid, which allows achieving high selectivity for some cancers. Similarly to antibodies, folic acid raises the affinity of particles to pathological tissues, thus increasing their accumulation in the target area. Due to the augmented expression of the folate receptor in the cancer cells, folic acid conjugates penetrate more easily through the cell membrane of folate-overexpressing cells. On this basis, Feng et al. designed a new photosensitizer—folic acid-conjugated silica-coated titanium dioxide [36]. The biocompatibility of the conjugate system was assessed in two cell lines: fibroblast cells (L929) and the human nasopharyngeal epidermoid cancer (KB) cells. After 24 h incubation, significantly better permeability of folic acid conjugated silica-coated TiO_2_ to L929 and KB cells was observed. Firstly, the effect of UV (365 nm) radiation on cells was tested and found non-toxic. The photosensitizer was applied at the concentration range from 12.5 up to 100 μg/mL, with the best effect on the KB cell viability reduction up to 57% at the highest concentration applied. Fluorescence tests revealed that cells absorbed significantly less neat TiO_2_ NPs (P25) than the conjugated system. Also, higher mortality of cells treated with the conjugated system than neat TiO_2_ alone showed the contribution of folate receptors in its internalization [36]. More insight into the influence of SiO_2_ shell on the activity of TiO_2_ NPs gave the study conducted by the same group. In the paper, there were presented data on the silica coating thickness on TiO_2_ NPs for effective photodynamic therapy [43]. The effect of the thickness of the silica shell on the photodynamic activity of TiO_2_ NPs, cytotoxicity, and photo-killing ability was unambiguously confirmed. On the one hand, it was found that the increase in the thickness of the silica shell allows better penetration of NPs through the cell membrane, reducing significantly, on the other hand, the photoactivity of the photosensitizer. Researchers were looking for the most optimal silica shell thickness guaranteeing maximum photo-killing efficiency while ensuring the best possible cytocompatibility. After a series of experiments on L929 cells, it turned out that the 5.5 nm SiO_2_-layer thickness seems to be optimal for the complete preservation of the photodynamic properties of TiO_2_ NPs and the improvement of their biocompatibility.

The application of TiO_2_ in PDT concerns its various forms, especially composites and hybrids, with some perspectives to use also in the area of wound healing application and management [44]. Archana et al. obtained and characterized blends of chitosan, poly(N-vinylpyrrolidone) and TiO_2_ by infrared spectroscopy, thermogravimetric analysis, transmission electron microscopy, and scanning electron microscopy [45]. The mechanical properties of composite material indicated that the addition of TiO_2_ NPs increases the strength of nanocomposite. The nanocomposite dressing revealed excellent antimicrobial efficacy and good biocompatibility against NIH3T3 and L929 fibroblast cells. Also, the material triggered accelerated healing of open excision type wounds in an albino rat model [45].

❖ **To sum up**

○ The applications of neat titania NPs in photodynamic therapy are limited by the necessity to use UV light of very low tissue penetration, and harmful impact on the human body.
○ Neat TiO_2_ NPs and in combination with various molecules, antibodies, or polymers revealed interesting photocytotoxicity against cancer cells and microbes, thus unveiling potential for PDT.
○ The SiO_2_ shell influences the activity of TiO_2_ NPs in photodynamic therapy. Only the optimal SiO_2_-layer thickness guarantees optimal preservation of the photodynamic properties of TiO_2_ NPs as well as the improvement of their biocompatibility.
○ TiO_2_ and its composites with chitosan, poly(N-vinylpyrrolidone) can broaden the current PDT applications towards the area of wound healing management.

## 6. Doping of TiO_2_ Nanoparticles with Inorganic Compounds and Carbon-Based Nanomaterials

The energy necessary for titanium dioxide NPs excitation is high, which is the result of a wide bandgap. Therefore, only UV light bears sufficient energy to excite titania [37]. Serious problem related to colloidal TiO_2_ NPs is their pH-dependent tendency to form agglomerates, which reduces their photoreactivity and decreases the functional surface area [46]. An additional limitation for the broader use of neat TiO_2_ NPs is their insufficient selectivity and the lack of cell-specific accumulation. The addition of inorganic compounds to the TiO_2_ NPs structure during their preparation or creation of defects in the structure of already prepared TiO_2_ NPs is defined as doping. This process narrows the bandgap in the TiO_2_ NPs structure and lowers the activation energy. Titania may be doped with a series of molecules, including organic, inorganic—both metals and nonmetals. Many studies with TiO_2_ NPs have been performed so far to maximize their visible light absorption. For example, it was found that doping or modification of the TiO_2_ NP surface leads to a shift of the absorption maxima towards longer wavelengths, thus increasing the depth of tissue penetration [43].

One way to improve the effectiveness of TiO_2_-based photodynamic therapy is modifying titania to the so-called black TiO_2_ NPs by reduction of the particles and inducing the formation of Ti^3+^ ions on the TiO_2_ surface. Such black TiO_2_ NPs were obtained by Ni et al., starting from the commercially available titanium dioxide (P25, 71% anatase and 29% rutile) powder and following a facile calcination method combined with an *in situ* controllable solid-state reaction method [7]. In this process, according to X-ray photoelectron spectroscopy (XPS) and UV-Vis DRS measurements, the existence of Ti^3+^ defects and oxygen vacancies in the black TiO_2_ was confirmed. Researchers used black TiO_2_ NPs as a near-infrared light-triggered PDT photosensitizer with a maximum absorbance of 808 nm on human bladder cancer cell line (T24). Bladder cancer cells were incubated with the photosensitizing NPs and then irradiated with laser at 808 nm for 0-7 min. As expected, an increase in the concentration of the photosensitizer correlated with an increase in anticancer activity. Minimal cell viability (54.32%) was observed at a concentration of 500 μg/mL, and the exposure time of 7 min. As noted by the authors, the applied black TiO_2_ NPs can be considered as an excellent photosensitizer due to their flexible-dose and very good anti-cancer effect. What is more, black TiO_2_ NPs on the contrary to non-doped TiO_2_ NPs were the most active in visible light and NIR.

Intensive work is currently underway on various combinations of TiO_2_ NPs with other inorganic elements and compounds to improve their photochemical properties. Kayani et al. developed cerium-doped (Ce-doped) TiO_2_ thin films synthesized by the sol-gel dip-coating route [47]. The band gap of the Ce doped TiO_2_ slightly decreased from ~3.93 eV to ~3.79 eV with an increase in Ce doping percentage. The obtained NPs showed favorable changes in the area of ferromagnetic sensitivity that can be correlated with the increase in cerium concentration. Unfortunately, these changes did not translate into any biological activity of the formulation. Cerium-doped TiO_2_ NPs were tested on *Pseudomonas aeruginosa*, *Escherichia coli*, *Klebsiella pneumoniae,* and *Staphylococcus aureus.* Following the results, the NPs did not reveal any photodynamic activity even when the amount of the compound increased (16 mg/mL) in agar medium. This can be also associated with the problems that appeared during the measurements as the tested NPs precipitated in the agar and did not mix properly with the agar medium [47]. The anti-cancer activity of modified and unmodified titanium(IV) oxide NPs has also recently been studied by Shah et al. [48]. Their research clearly indicated that TiO_2_ nanoparticles stabilized with PEG (polyethylene glycol) reveal better photodynamic activity than the non-stabilized NPs. Their antitumor activity was assessed *in vitro* on human cervical cells (HeLa) and human skin cancer cells (HT144). The reported results support the further development of nanomaterials based on the combination of titanium dioxide with polymers. The authors also extended the research by analyzing the activity of modified TiO_2_ NPs. What is essential, doping of the metal (cobalt) and non-metal (nitrogen) onto TiO_2_ nanocrystals allowed the photoactivation of doped-TiO_2_ NPs in the visible/near-infrared region. Paradoxically, however, despite an improvement in their photochemical parameters, anti-tumor activity declined. The authors associate it with the so-called downregulated ROS production or reduced uptake of conjugates by cancer cells [48]. In another study, performed by Zeni et al. [49], nitrogen-doped TiO_2_ NPs were applied *in vitro* on murine melanoma cell line (B16-F10) and fibroblasts (NIH 3T3). The nitrogen-doped TiO_2_ NPs were prepared following a modified hydrogen peroxide sol-gel process with triethylamine as a nitrogen precursor. Moreover, X-ray diffraction (XRD) measurements confirmed that all TiO_2_ and N–TiO_2_ samples consisted of an anatase crystalline phase without any trace of rutile. The nitrogen-doped TiO_2_ NPs revealed higher absorbance in the visible region than the neat ones. The use of modified titanium(IV) oxide at a concentration of 0.5 mg/mL resulted in the death of up to 93% melanoma cells under UV irradiation treatment and caused an increase in expression of the pro-apoptotic BAX gene. A comparison of both results may indicate that the sensitivity of cancer cells to modified NPs may vary significantly depending on the type of cancer [49].

An example of an interesting composite obtained by doping of the TiO_2_ NPs with carbon-based nanomaterials was reported by Shang et al. [50]. They used TiO_2_ NPs doped with reduced graphene oxide (RGO-TiO_2_) in 10%-50% m/m ratio. The novel composite was obtained by the hydrothermal reduction method and then characterized by XRD, infrared spectroscopy, transmission electron microscopy, Brunauer-Emmett-Teller (BET) surface area analysis, UV-Vis spectroscopy, and XPS. Comparative analysis proved that RGO-containing NPs, when the modified proportion was 0.2 (RGO:TiO_2_), were more active against human hepatocellular carcinoma (HepG2) cancer cells than neat TiO_2_ NPs. In the study, either UVA (365 nm) or visible light (420 nm) were used. On the one hand, only at a long-term incubation with RGO-TiO_2_ NPs, the material revealed dark toxicity in concentration range 0-500 μg/mL which resulted in up to 25% viability decrease, but on the other hand, it was less toxic in the dark than TiO_2_ NPs alone. The treated cells died turning on apoptosis pathway as a result of increased oxidative stress. The effects observed in tested cells were as follows: a marked decrease in the ratio of the super-coiled DNA indicating DNA oxidative damage, mitochondrial membrane potential disruption, as well as an increased intracellular calcium concentration. Very similar data in terms of observed cytotoxicity of the carbon material were obtained in the study performed by Ismail et al. [51]. They explored the antiproliferative activity of – among others—TiO_2_-NPs (P25), titanium dioxide nanotubes (TiO_2_-NTs), and ZnO-NPs/TiO_2_-NTs nanocomposite under UV irradiation and received completely different results to the above-described. In this study, human liver adenocarcinoma cells HepG2 were incubated for 48 h with the plethora of various zinc oxide NPs, metal-doped zinc oxide NPs, silica-coated zinc oxide NPs, as well as the above-mentioned titania-containing materials at five different concentrations (6.25, 12.5, 25, 50 and 100 μg/mL). The plates were then irradiated for 3 min with light 320-400 nm (UV light). Interestingly, it was found that the metal-doped ZnO-NPs can induce an antiproliferative effect on HepG2 cells under UV-irradiation due to the generation of ROS. Surprisingly, none of the tested NPs containing TiO_2_ showed statistically significant anti-proliferative activity. The authors indicate that the problem may result from the limited exposure time of NPs to UV light. Compared to the study mentioned earlier, the low activity of NPs with TiO_2_ might result from their low accumulation in tissues due to the unmodified surface of the NPs and thus their higher agglomeration [51].

❖ **To sum up**

○ Doping or modification of TiO_2_ NPs “turns on” their excitation possibilities by visible light and increases their activity in photodynamic activity study.
○ The combinations of TiO_2_ with inorganic dopants and carbon-based nanomaterials modifying its photochemical properties seem to be an alternative not only to neat TiO_2,_ but also to conventional photosensitizers in PDT.
○ The combinations of TiO_2_ with inorganic dopants and carbon-based nanomaterials were studied towards antimicrobial and anticancer PDT.

## 7. Modifications of TiO_2_ Nanoparticles with Photosensitizers Aiming to Improve Their Optical and Biological Properties

Metallic nanoparticles, based on gold, silver, and titanium, reveal their photodynamic activity in many *in vitro* and *in vivo* biological studies. The development of PDT studies based on the combination of nanoparticles or quantum dots with commonly used photosensitizers, such as phthalocyanines, porphyrins, and other dyes, is becoming more popular. NPs appear to be also suitable carriers for targeted therapy. The use of proper drug delivery systems for photosensitizers allows performing PDT in specific tissues [52,53].

Because of the shortcomings of neat titania NPs, mostly due to their absorption of only short UV wavelengths and aggregation in water media, they have been modified with a plethora of inorganic and organic dopants. Among organic dyes most often utilized for combining with TiO_2,_ are porphyrins and phthalocyanines. Such hybrid materials were numerously tested for their use as catalysts for visible-light biomedical and environmental photocatalysis in photovoltaics for the preparation of dye-sensitized solar cells (DSSC) as well as photosensitizers for PDT [54,55,56,57]. The selected TiO_2_ NPs combined with photosensitizers discussed in this chapter are summarized in Table 1.

### 7.1. TiO_2_ Nanoparticles Combined with Phthalocyanines

**Figure 4:**
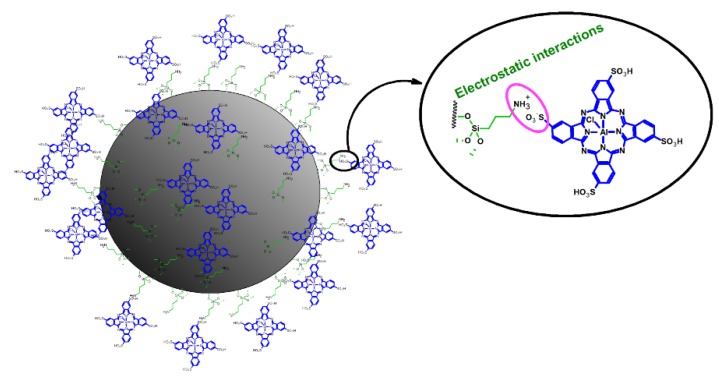
Aluminum tetrasulfonatedphthalocyanine chloride linked to nitrogen-doped anatase TiO_2_ nanoparticles by electrostatic interactions (based on [58]).

An excellent example of a combination of TiO_2_ NPs and phthalocyanine was presented by Pan et al., who linked aluminum(III) tetrasulfonatedphthalocyanine chloride (TSAlClPc) to nitrogen-doped anatase TiO_2_ NPs by electrostatic interactions (Figure 4) [58]. The hybrid material was characterized by Zeta potential measurements, transmission electron microscopy, and UV–Vis absorption spectroscopy. The cellular uptake, intracellular distribution, cytotoxicity and the photokilling effect of the NPs were studied on human epithelial cervical cancer cells (HeLa) and human nasopharyngeal carcinoma cells (KB). On the one hand, the phthalocyanine selected for the study is known to present high absorption in the red light region, but on the other hand, its use is limited due to reduced cell permeability. The absorption spectrum of hybrid material demonstrated features of both components with absorption maxima in the red region of the spectrum and UV region, which resulted in higher production of reactive oxygen species under visible light irradiation. Moreover, both cellular uptake and intracellular distribution of phthalocyanine were remarkably improved by its nitrogen-doped TiO_2_ carrier. In the biological study, HeLa or KB cancer cells were incubated for 1 h with the photosensitizer at the concentrations from the range 4.7 to 37.6 μg/mL for one hour and then immediately irradiated with a light 420-800 nm (15 J/cm^2^). It turned out that hybrid material at the concentration of 21.88 μg/mL killed up to 86% of tumor cells. It means that the photokilling efficiency of hybrid material was over ten times higher and ROS production was 2.6-fold better than that measured for the studied phthalocyanine alone [58]. Also, in 2017 Pan et al. published the results of a very similar study, in which they tested the activity of TSAlClPc, non-doped and nitrogen-doped TiO_2_ NPs as well as their conjugates [59]. Photosensitizers were applied at concentrations in the range of 5 up to 20 mg/mL for the HeLa tumor cells study. The absorbance and photokilling effect on HeLa cells were studied upon visible light irradiation of different regions of 420-800 nm (15.9 J/cm^2^) and 420-575 nm (7.5 J/cm^2^). The best photocytotoxic activity of the nitrogen-doped TiO_2_-TSAlClPc system was measured under irradiation of 420-800 nm when over 85% of the cancer cells were killed. Non-doped TiO_2_-TSAlClPc NPs were found less active as they killed slightly more than 70% of the HeLa cells, while TSAlClPc alone presented only weak photokilling effect with more than 83% of cells surviving. Moreover, it was noticed that the nitrogen-doping of NPs can significantly increase photodynamic activity, as it greatly enhances the formation of singlet oxygen (^1^O_2_) and superoxide anion radicals, whereas it suppresses the generation of hydroxyl radicals [59].

A new developing part of medicine is the so-called theranostics—a combination of diagnosis and therapy. Theranostics uses specific pathways in the body to achieve a specific molecular target, e.g., a specific receptor on cancer cells. Also, theranostics is the basis for targeted therapy, the part of which may be anti-cancer PDT [70]. Yurt et al. used in their study an unsymmetrical monocarboxylic derivative of zinc(II) phthalocyanine (MCZnPc) and MCZnPc anchored onto TiO_2_ NPs—labeled with ^131^I to assess their potential in therapy and diagnosis of selected cancers. MCZnPc and MCZnPc-TiO_2_ NPs were tested for their antitumor activity against mouse mammary carcinoma (EMT6) and human cervical adenocarcinoma (HeLa) cells [60]. After a three-hour incubation period in the dark, the cell cultures with photosensitizers were irradiated with light at a 684 nm wavelength. This study has also focused on the cellular uptake of the radiolabeled MCZnPc and radiolabeled MCZnPc-TiO_2_. The results demonstrated higher cellular uptake for the labeled MCPc-TiO_2_ NPs. Therefore, they can be considered good candidates for nuclear imaging and hence for theranostic applications against breast and cervical tumors. It was found that the photokilling effect of MCZnPc and MCZnPc-TiO_2_ conjugates against both EMT6 and HeLa cells increases proportionally to the concentration of photosensitizer and light intensity topping at over a 80% decrease in cell viabilities. MCZnPc-TiO_2_ caused significant phototoxicity in EMT6 cell lines at 1.57 and 3.13 μM with 30 J/cm^2^ and 6.25 μM at 60 J/cm^2^. The photocytotoxicity in HeLa cell lines was the highest for MCZnPc-TiO_2_ at 6.25 μM with 60 J/cm^2^, but MCZnPc was more photocytotoxic at 3.13 μM with 90 J/cm^2^ [60].

The limitation of the possible side effects and the increase of tissue specificity of phthalocyanines, which are generally perceived as relatively non-toxic compounds, has been considered as a goal of many studies [71]. For this purpose Yurt et al. anchored MCZnPc with pure TiO_2_ NPs [72]. The obtained material was tested as a potential agent for photodynamic therapy/photodynamic diagnosis on human healthy lung fibroblast cells (WI38) as well as selected cancer cell lines—hepatocellular carcinoma (HepG2) and colorectal adenocarcinoma (HT29). The MCZnPc solution or MCZnPc-TiO_2_ dispersion in DMSO were added to the tumor cell suspension and incubated in the dark for 3 h, after which the cells were irradiated. The study confirmed the assumption that such a combination of MCZnPc and TiO_2_ significantly reduces the toxicity of the photosensitizer. The hybrid ZnPc-TiO_2_ material revealed higher phototoxicity than the studied phthalocyanine alone in colon tumor treatment. Parallel, the results of the study proved that MCZnPc-TiO_2_ showed a stronger photokilling effect on the HepG2 than MCZnPc alone. It was also found that the photokilling effect increased with the concentration of the photosensitizer and the light intensity. The maximum effect was achieved for MCZnPc-TiO_2_ at a concentration of 6.25 mM and a light intensity of 90 J/cm^2^ with killing efficiency over 73% on the HepG2 cells. Also, MCZnPc-TiO_2_ was radiolabeled with ^131^I radioisotope, and the uptake of ^131^IMCZnPc-TiO_2_ in the cell lines was studied for potential applications as a bifunctional agent in nuclear imaging and PDT. According to the results of intracellular uptake, the high target/non-target tissue ratio of ^131^I labeled MCZnPc-TiO_2_ can be applied as a nuclear imaging agent for hepatocellular cancer [72].

The modified TiO_2_ NPs also reveal an interesting potential for antimicrobial photodynamic chemotherapy (PACT). Many of the metals, including gold or silver, have been used so far for the preparation of NPs, and demonstrate relatively strong antibacterial properties. Titanium compounds, especially titanium dioxide, were also used as a bactericide, as evidenced by numerous *in vitro* studies on various bacteria including both Gram-positive and Gram-negative strains [56,73,74,75]. The antibacterial properties of titanium dioxide can be enhanced by exposure to UV light. Currently, there are several ongoing studies focused on photocytotoxicity of hybrid materials composed of phthalocyanines, porphyrazines or chlorines bound to titanium dioxide. Two recently published studies indicate that the combination of TiO_2_ with phthalocyanines and subphthalocyanines raises the effectiveness of antimicrobial photodynamic therapy. On the one hand, Tunçel et al. showed that the application of Zn(II) phthalocyanine with (4-carboxyphenyl)ethynyl moieties alone or after integration with TiO_2_ NPs leads to very similar photocytotoxicity results [61]. On the other hand, the cellular uptake of these molecules was substantially lower when phthalocyanine was combined with titanium dioxide. The authors associate this fact with the increase of molecular weight decreasing the efficiency of photosensitizer penetration through the bacterial wall. In the same research group, Ozturk et al. proved that the combination of subphthalocyanine with titanium NPs increases their activity against *S. aureus* and *E. coli* [62]. It should be emphasized that in both cases the conjugates required lower light intensity to achieve a bactericidal effect. The viability of *S. aureus* and *E. coli* strains after irradiation was dependent upon both light doses and the compounds used in the treatment. Here, however, the cell uptake of the compounds was higher in the case of pure phthalocyanine. It is worth noting that a bactericidal effect against *S. aureus* of subphthalocyanine was observed at 24 J/cm^2^, whereas the subphthalocyanine-TiO_2_ hybrid material was active at 16 and 24 J/cm^2^ of light doses, which suggests the increased photoactivity of the material as compared to its components alone. Moreover, both subphthalocyanine and subphthalocyanine-TiO_2_ hybrid material were active against Gram-negative strain *E. coli* at 30 J/cm^2^ of light dose [61,62]. Mantareva et al. studied the photodynamic activity of Zn(II) phthalocyanine salt containing 3-dodecylpyridyloxy moieties (ZnPcDo) adsorbed on TiO_2_ anatase NPs *in vivo* against methicillin-resistant *Staphylococcus aureus* (MRSA) and *Salmonella enteritidis* [63]. The authors assessed the use of 346 nm light, 643 nm light, or both light sources simultaneously. The ZnPcDo-TiO_2_ hybrid material demonstrated high activity against MRSA bacteria after exposure to red light (2 logs inactivation), but no antimicrobial activity was observed after irradiation with UVA alone. Tests on *S. enteritidis* strain revealed that only the ZnPcDo-TiO_2_ combination was active after UVA and LED exposure. As pointed out by authors, irradiation with two sources at the same time does not significantly increase the photodynamic activity of the photosensitizer. The practical application of the novel hybrid material was related to photoinactivation of pathogenic bacteria in wastewater [63].

Antimicrobial photodynamic therapy can be used not only against bacteria or fungi, but also against parasites. Lopez et al. studied the photocytotoxicity of zinc(II) phthalocyanine (ZnPc), nano-TiO_2_, and ZnPc-TiO_2_ composite, against a panel of tumor and non-tumor mammalian cells, including the African green monkey epithelial cells (Vero cells, ATCC), human hepatocellular liver carcinoma cells (HepG2, ATCC), human acute monocytic leukemia cell line THP-1 (ATCC), and a primary culture of human-derived fibroblasts (HDFs) and on promastigote forms of Leishmania parasites [64]. Neat TiO_2_ NPs under visible light irradiation were not phototoxic for the cells, whereas ZnPc was photocytotoxic for all the studied cells and Leishmania parasites. Mammalian cells were incubated with TiO_2_, ZnPc-TiO_2_, or ZnPc for 24 h and then illuminated with light (either 670 nm or in the 597-752 nm range). The growth was microscopically assessed by counting parasite numbers. Unfortunately, contrary to expectations, neither TiO_2_ NPs nor ZnPc-TiO_2_ caused phototoxic effect against tested *L. chagasi* or *L. panamensis* promastigotes. However, ZnPc-TiO_2_ was active against tumor and non-tumor mammalian cells, but less than the pure ZnPc. Moreover, ZnPc-TiO_2_ was internalized by the cells at a lower level than ZnPc. Both ZnPc-TiO_2_ and ZnPc were localized in mitochondrial cytoplasm [64].

A study in which phthalocyanine was deposited on TiO_2_ nanopore thin films, can be indicated as the further development of TiO_2_ applications in photodynamic therapy. Perillo et al. used this method to prepare a potential photosensitizer containing copper tetracarboxyphthalocyanines (TcPcCu) active against MRSA [65]. The suspension of bacteria and photosensitizer was irradiated with visible light. A sample containing only TiO_2_ thin film showed no differences as compared to the control. However, the TiO_2_/PcTcCu thin film sample reduced the development of MRSA by 81.5% [65].

### 7.2. TiO_2_ Nanoparticles Combined with Porphyrins, Chlorins and Methylene Blue

**Figure 5:**
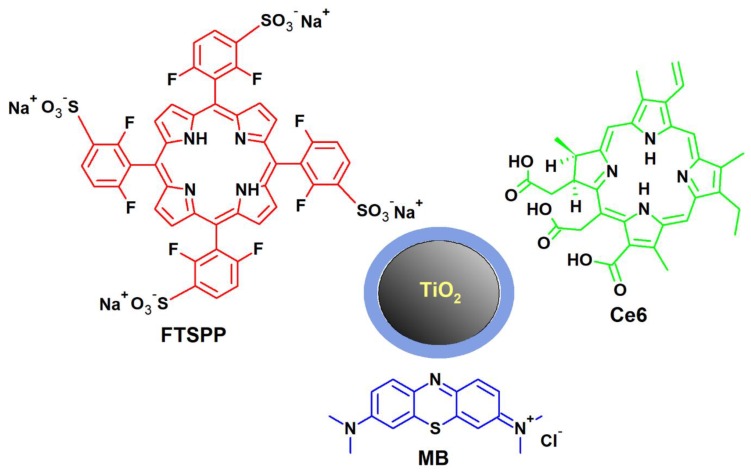
Selected photosensitizers combined with titanium dioxide nanoparticles; 5,10,15,20-tetrakis(2,6-difluoro-3-sulfophenyl)porphyrin (FTSPP), Chlorin e6 (Ce6), methylene blue (MB).

Porphyrins doped on TiO_2_ NPs were studied in terms of their potential applications in photodynamic antimicrobial therapy by Sułek et al. (Figure 5) [57]. They received very promising results combining titanium dioxide with fluorinated porphyrins, 5,10,15,20-tetrakis(2,6-difluoro-3-sulfophenyl)porphyrin (FTSPP) and its zinc(II) derivative [57]. The combination of TiO_2_ NPs with the obtained FTSPP halogen derivatives improved the overall properties of both compounds. In the study, the influence of conjugates on representative Gram-positive (*S. aureus*) and Gram-negative (*E. coli*) bacteria was assessed. The combination of fluorinated porphyrins and TiO_2_ NPs after two hours of exposure to visible light (1 J/cm^2^ at 420 nm or 10 J/cm^2^ at 530 nm) led to spectacular 7 logs reduction in colony-forming units. The activity of conjugates against Gram-negative bacteria was significantly lower, which is typical for PACT. It is worth noting that the addition of KI (50 mM) transformed the hybrid materials into effective antimicrobial photosensitizers able to efficiently inactivate Gram-negative bacteria. Remarkably higher activity of hybrid material towards Gram-positive bacteria was related to the specificity of the cell wall structure of these bacteria. Unfortunately, the additional outer-membrane that contains lipopolysaccharide present in the structure of Gram-negative bacteria, decreases membrane permeability for lipophilic compounds and impedes the penetration of reactive oxygen to the target structures in the cell, thereby limiting the effectiveness of the photosensitizer [57].

The application of PDT is not only limited to the treatment of cancer and bacterial infections, but it is also becoming more popular for the treatment of other diseases. The first generation of porphyrins has been applied in clinical practice for several years. Although the quality of porphyrin photosensitizers considerably improved over the years, mainly due to the development of the second- and third-generation photosensitizers, the problem of efficient distribution and selectivity of these compounds in the body has still not been resolved. The implementation of the therapy with the use of porphyrins and derivatives, as well as various NPs, was extended and also applied in rheumatoid arthritis, atherosclerosis, macular degeneration, and diabetes mellitus [76].

Many attempts have been made to combine porphyrins with nanoparticles, including TiO_2_ NPs, aiming to obtain an effective formulation. Zhao et al. designed a nanocomposite from the water-soluble TSPP and TiO_2_ nanowhiskers as a potential agent in the theranostics and PDT of rheumatoid arthritis [66]. Rheumatoid arthritis is an autoimmune disease and is usually treated with TNF-α blockers, minocycline, azathioprine, and non-steroidal anti-inflammatory drugs. The hybrid material was tested on rats and mice with artificially collagen-induced arthritis. After injection of aqueous TSPP-TiO_2_ suspensions, for fluorescence imaging, the animals were treated using a special device (IVIS Lumina XRMS Series III) with excitation wavelength at 520 nm and emission wavelength at 620 nm. The measurements were conducted from the start of the study until the day of the initial clinical symptoms (day 28). For the first time, very strong fluorescence was observed on day 16, when the first clear clinical symptoms had not yet occurred. Importantly, during the test, only sick joints showed a clear and intense fluorescence, while the muscles showed poor fluorescence—this indicates that the photosensitizer accumulated selectively in areas of inflammation. The properties of the photosensitizer allow it to be used as a selective bio-imaging agent at the early (sub-clinical) stage of the disease without the risk of complications. Singlet oxygen quantum yields were determined using a time-resolved Nd:YAG laser set-up with excitation at 532 nm and a liquid N_2_ cooled Ge photodetector. The nanowhiskers solution in the PDT experiment on rats revealed an ameliorating effect on the rheumatoid arthritis by decreasing significantly the IL-17 and TNF-α level in blood serum. In addition, fluorescent imaging was helpful in the diagnosis of the rheumatoid arthritis disease in subclinical stages and to bio-mark the rheumatoid arthritis affected joint. These findings are of a very important value, as rheumatoid arthritis is one of the major age-related diseases with roughly 1% of the population suffering from this disease, which is predicted to rise [66,77].

In another experiment, Rehman et al. utilized photodynamic therapy in the treatment of diabetes mellitus [67]. It is a severe metabolic disease that occurs in two types: type I, so-called insulin-dependent, and type II, non-insulin-dependent. Although PDT has been used for the treatment of many cancer diseases, its use in the therapy of metabolic diseases is still considered innovative. According to the procedure, each treated mouse was injected TSPP-TiO_2_ and irradiated for 1 h with visible light (500–550 nm), every day for a week. The results indicated that such an approach was effective only in the case of non-insulin-dependent diabetes mellitus, as two hours after the treatment, a reduction in sugar levels by up to 33% was observed. The type II diabetes mellitus mouse model response to photoactivated nanocomposites was observed in lowering the blood glucose level, which could be due to ROS and ^1^O_2_ generation during PDT. As a consequence, it could influence cellular uptake and metabolism of the glucose with the help of insulin. As the authors pointed out, this could also be related to a decrease in the number of mitochondria in visceral adipocytes, leading to a reduction of white fat content in the body and greater sensitivity to insulin.

As already mentioned above, TiO_2_ NPs allow to significantly increase the selectivity of porphyrin and chlorin photosensitizers and reduce their adverse effects. The properties of TiO_2_ NPs allow the use of even potentially more toxic photosensitizers. Therefore, scientists have been trying to combine them with photosensitizers belonging to other chemical groups, including chlorins and methylene blue. In one of the studies, TiO_2_ NPs were conjugated with the photosensitizer Chlorin e6 (Ce6) (Figure 5) [68]. TiO_2_ NPs were modified by adding layers consisting of two silane reagents (3-aminopropyl) triethoxysilane (APTES) and tetraethyl orthosilicate (PEGylated NPs: TiO_2_ @ 4 Si-Ce6-PEG). Also, NPs modified only by APTES (APTES-modified NPs: TiO_2_-APTES-Ce6) were prepared. Ce6 was covalently bound to TiO_2_ NPs (P25 containing 75% anatase and 25% rutile) through an amide bond. The photocytotoxicity of hybrid NPs was assessed on the glioblastoma cells (U87), after irradiation with a 652 nm light at fluence rate of 10 J/cm^2^. Nanoparticles modified with APTES alone demonstrated higher photodynamic activity in comparison to PEGylated core-shell structured NPs. In the first case, the unique photokilling effect of the photosensitizer was observed at the highest concentration and the cancer cell viability was decreased by 89% after the visible light illumination in the presence of 200 μg/mL of TiO_2_-APTES-Ce6 NPs, which held a concentration of 0.22 μM of Ce6. A similar effect was obtained using Ce6 alone, but at a much higher concentration (10 μM). This indicated that TiO_2_-APTES-Ce6 conjugates led to an increase in cellular uptake of the photosensitizer [68].

Tuchina et al. performed the photocytotoxicity study with one of the oldest photosensitizers used in the laboratory and medical practice—methylene blue (MB) (Figure 5) [69]. They investigated the antimicrobial photodynamic activity of a mixture of two individuals—MB and TiO_2_ NPs against *S. aureus*, *E. coli*, and *Candida albicans*. Suspensions of bacteria or fungi together with the photosensitizer were incubated in the dark for 10 min and then irradiated with two LED lamps simultaneously (405 and 625 nm). The mixture of both PSs and irradiation with red and blue light simultaneously, reduced the number of *S. aureus* cells by up to 90%. Almost identical results were obtained using a combination of photosensitizers against *C. albicans*. Curiously, almost no activity against *E. coli* was observed.

❖ **To sum up**

○ The combination of photosensitizers with TiO_2_ nanoparticles can be beneficial for the effectiveness of PDT and can reduce the side effects of chemotherapy.
○ Among organic dyes, most often utilized for combining with TiO_2_ are porphyrins and phthalocyanines, which were numerously applied as photosensitizers for PDT.
○ The nitrogen-doping of TiO_2_ NPs combined with phthalocyanines can significantly increase the efficacy of photodynamic activity, as it greatly enhances the formation of singlet oxygen and superoxide anion radicals, whereas it suppresses the generation of hydroxyl radicals.
○ Phthalocyanines anchored onto TiO_2_ NPs and labeled with ^131^I were assessed for PDT diagnosis of selected cancers.
○ Hybrid materials composed of phthalocyanines, porphyrazines, or chlorines bound to TiO_2_ were studied in terms of their effectiveness in antimicrobial PDT against bacteria, fungi, and parasites.
○ The combination of fluorinated porphyrins and TiO_2_ NPs after exposure to visible light revealed 7 logs reduction in colony-forming units of *Staphylococcus aureus*. The addition of KI transformed the hybrid materials into effective antimicrobial photosensitizers able to efficiently inactivate Gram-negative bacteria.
○ The application of PDT with the use of porphyrins and their derivatives as well as various NPs was extended to rheumatoid arthritis, atherosclerosis, macular degeneration, and diabetes mellitus.
○ The combination of methylene blue with TiO_2_ NPs irradiated with light sources simultaneously (405 and 625 nm) reduced the number of *S. aureus* cells by up to 90%. Almost identical results were obtained using a combination of photosensitizers against *C. albicans*.

## 8. TiO_2_ Nanoparticles As a Vehicle for Chemotherapeutics

**Figure 6:**
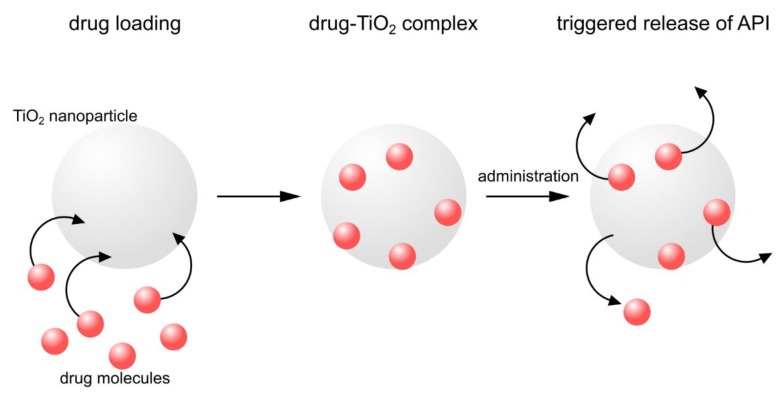
Simplified mechanism of titanium(IV) oxide as drug delivery vehicle.

Cancer is considered one of the most significant challenges for modern medicine. Despite the continuous development of modern cancer treatment methods, the first line of therapy is the surgical removal of tumor and/or radiotherapy. Chemotherapy is usually a complementary therapy, but it is limited by many factors. First of all, chemotherapeutics are extremely toxic to rapidly proliferating, both cancer and healthy, tissues in the human organism. Therefore, novel delivery systems that would increase the tissue specificity of the therapy and reduce the systemic effects are still being sought. Many chemotherapeutic agents are also ineffective because of the multidrug resistance (MDR) mechanism displayed by cancer cells and related to the overexpression of some members of the ABC superfamily of efflux transporters that treat the drug as a poison and remove it from the matrix [78,79]. Currently, one of the most commonly studied chemotherapeutic drug is doxorubicin (DOX). Although it provides many advantages for the therapy of various cancers, the use of doxorubicin is associated with adverse effects, out of which, the cardiotoxicity is the most severe and dangerous [80]. Potential solution to both problems may be the use of nanoparticles and the combination of chemotherapy with photodynamic therapy or photothermal therapy (Figure 6). Titania nanoparticles offer significant advantages in this field, enabling efficient delivery of the drug molecules, and thus better pharmacokinetics, and their targeted delivery [81,82].

Inorganic photosensitizers, including TiO_2_ NPs, similarly to organic photosensitizers, can be used for PDT, which was presented above. TiO_2_ NPs were investigated for PDT under the excitation of UV or visible light after doping with organic photosensitizers. Considering some limitations of UV light mainly related to harmful effects for human body accompanied by low penetration to tissues, it is worth exploring NIR-triggered inorganic photosensitizers for the non-invasive PDT in deep tissues. The rare-earth-doped nanoparticles converting NIR into UV light, NIR-triggered PDT of TiO_2_ inorganic photosensitizers can be obtained by coupling two different NPs, including up-conversion NPs (UC NPs) and TiO_2_ NPs. UC NPs can be coated by TiO_2_ inorganic photosensitizers to form a core-shell structure or combined to form UC/TiO_2_ nanocomposite with a non-core-shell structure. The construction of nanocomposite seems to be more beneficial as such a system can be applied for the chemotherapy and NIR-triggered inorganic PDT. Improvement of the DOX targeting to the cancer cells may be achieved by selective release of the drug in the desired site of action. This would then result not only in lowered toxicity of the anticancer agent, but also in increased concentration of the drug in cancer cells, enabling lower doses to be used and reducing the occurrence of adverse effects. Selected TiO_2_ NPs combined with doxorubicin discussed in this chapter are summarized in Table 2.

The role of TiO_2_-based nanomaterials in confinement of adverse toxic effects of DOX was presented by Zeng et al. [88]. The scientists designed folic acid (FA)-targeted NaYF_4_:Yb/Tm-TiO_2_ nanocomposites and loaded them with DOX (FA-NPs-DOX) for near-infrared (NIR)-triggered inorganic PDT and enhanced chemotherapy to overcome MDR of breast cancers both *in vitro* and *in vivo*. An *in vivo* study was performed on female Balb/c nude mice bearing DOX-sensitive human breast cancer cells MCF-7 or DOX-resistant MCF-7/ADR tumors. The FA-NPs and FA-NPs-DOX-treated groups were additionally irradiated for 10 min with laser light (980 nm) with a power density of 500 mW/cm^2^. Mice bearing both types of tumor were more sensitive to FA-NPs-DOX conjugates compared to free DOX, demonstrating the excellent efficacy of FA-NP-DOX nanocomposites for DOX-resistant tumors. It is essential that the viabilities of DOX-resistant MCF-7/ADR cells decreased from 71.1% to 17.6%, whereas the tumor inhibition rate of MCF-7/ADR tumor-bearing nude mice increased from 6.41% to 96.74%, compared with free DOX. Also, treatment with FA-NPs alone gave quite good results (inhibition at 83.62% and 76.86%). In both cases, irradiation played a significant role, substantially increasing inhibitory effect (almost doubling it in both cases). In a study by Flak et al., TiO_2_-based hybrids combined with zinc(II) phthalocyanine (ZnPc@TiO_2_) and folic acid (FA/ZnPc@TiO_2_) were studied as the delivery system for doxorubicin [83]. Their research confirmed that DOX could be easily applied to these structures and released due to electrostatic interactions. Furthermore, cytotoxic studies demonstrated that these nanostructures are selectively captured by cancer cells. Cellular uptake was estimated based on monitoring of the concentration by fluorescent confocal microscopy in 2D and 3D cell cultures. DOX-loaded photosensitizers accumulated more in HeLa cells than in normal fibroblasts (MSU-1.1). It was found that DOX-loaded hybrid nanostructures were significantly more cytotoxic than non-loaded ones. Moreover, the cytotoxic effect of DOX-loaded hybrid nanostructures was even more severe upon the near-visible irradiation with the viable cell fraction below 2% [83].

Similar effects confirming the decreased cytotoxicity of doxorubicin-loaded on upconverting nanoparticles TiO_2_ were presented by Chen et al. [84] and Tong et al. [89]. In the experiment, Chen et al. obtained special nanoplatforms (NaYF_4_:Yb,Tm@NaYF_4_) coated with hollow mesoporous TiO_2_ (UCNPs@mHTiO_2_). Toxicity was tested on the HeLa cell culture irradiated with NIR light at 980 nm, thus triggering the UV emission and in this way stimulating TiO_2_ towards the production of ROS. Porous and cavity structure of NPs allowed obtaining interesting antitumor drug DOX acid-enhanced drug release system, which was tested for intercellular specific chemotherapy. The synergistic effect of chemotherapy and PDT was presented within the study. Moreover, the excited energy from the higher-lying energy level (visible emission) of UCNPs was transferred to DOX via the luminescence resonance energy transfer mechanism. The intracellular drug release kinetics were studied by the recovery of the visible emission from UCNPs. The results showed a negligible toxicity (determined by MTT assay) of UCNPs@mHTiO_2_ on HeLa cells (the cell viability above 95.89%), while the combination of PDT with DOX-UCNPs@mHTiO_2_ exerted synergistic effect, causing death of almost 90% of cancer cells (the cell viability dropped to 11.26%). The optical bioimaging experiment revealed the potential application of the DOX-UCNPs@mHTiO_2_ in theranostics as it proved the recovery of the upconversion luminescence by the diffusion of most DOX molecules into the media. A different approach was utilized by Tong et al. They combined up-conversion nanoparticle core-mesoporous silica shell that was coated with titanium dioxide as a photosensitizer [89]. This system was then loaded with DOX molecules, which were trapped in silica pores by UV-sensitive linker o-nitrobenzyl derivative—linker TC. What is interesting, the UV emission induced the photodecomposition of TC linker, allowing the DOX drug release. Toxicity studies performed on HeLa cells showed slight dark toxicity of this system. In a further study, the nanocomposite mediated ROS after NIR-irradiation and released DOX. After illumination for five minutes with NIR light, cell viability decreased to 85%, 79%, and 77% after the treatment with UCNPs@mSiO_2_/TiO_2_, DOX-UCNPs@mSiO_2_-TC, and DOX-UCNPs@SiO_2_/TiO_2_-TC, respectively. Complex studies also confirmed that cells can quickly capture the prepared nano vehicle by endocytosis or macropinocytosis, which potentially increases the effectiveness of the therapy. Everything considered, DOX–UCNPs@mSiO2/TiO_2_-TC nano vehicle revealed usefulness for NIR light-sensitive chemo/photodynamic synergistic therapy.

Wang et al. used diamond-shaped TiO_2_ NPs for the delivery of DOX [85]. The prepared titanium dioxide nano bricks were functionalized with PEG chains and loaded with DOX molecules. Firstly, the material was found to exhibit pH responsiveness, as DOX was almost entirely released in acidic conditions, which are associated with cancer cells. Due to the porosity of the nanoparticles, the material could contain over 10% DOX. The material was then tested for its cytotoxicity on HepG2 cells, showing its non-cytotoxic nature (cell viability >80%) for empty nano bricks, whilst the viability for DOX-loaded NPs dropped to <10%. The subsequent *in vivo* test on Balb/c mice bearing H-22 tumors (murine hepatocellular carcinoma), showed the high activity of the nano bricks as drug delivery materials, because the mice treated with NPs were found to exhibit smaller tumor volumes as compared to the DOX-only treated group. Similar studies were conducted by Li et al. [86]. They tested the anti-tumor activity of the combination of doxorubicin with TiO_2_ NPs in human hepatocarcinoma therapy. In this case of *in vitro* tests, better results were obtained of synergistic action of both compounds. An increase in the biocompatibility of TiO_2_ and better photocatalytic activity was also observed [86].

One of the latest discoveries in the field of mixed chemotherapy and photodynamic therapy is the encapsulation of doxorubicin into "capsules" composed of Au-TiO_2_ NPs. This combination allows the drug for easy diffusion around the tumor due to the acidic environment of the tissue core. The released chemotherapeutic agent—DOX acts directly on the tumor tissue and reaches a higher concentration in it. On the other hand, the nanoparticles that form the shell take an active part in PDT by generating reactive oxygen species (under the influence of UV light). PDT based on the doped material of Au-TiO_2_ and Au-TiO_2_@DOX revealed cell-killing properties with its maximum achieved via a direct excitation process of the final product by laser light with a suitable wavelength of 500 nm. The synergistic combination of these substances resulted in a photocytotoxicity study *in vitro* on breast carcinoma cells MCF-7 in cancer-cell viability loss of up to 70%, according to MTT assay. The synergistic response of Au-TiO_2_@DOX via PDT was also confirmed in an *in vivo* study on the rat model [90]. As the development of gold-titania hybrids, Xu et al. elaborated a system of TiO_2_ nanotubes, that were functionalized with gold nanoparticles [87]. Ampicillin—an antibacterial drug—was attached via an alkylsilane to the system. Such hybrid system was tested *in vitro* on *E. coli* bacteria cultures. The designed activity was achieved—upon visible light irradiation. The drug molecules were cleaved without degradation and acted in an antibacterial manner. The researchers loaded antibiotic—ampicillin sodium in the lower part of the hydrophobic TiO_2_ nanotube stack and subsequently triggered visible-light-induced Au/TiO_2_ surface plasmon resonance release allowing the carrying out of antibacterial studies towards *E. coli*. When the system was not illuminated, the drug was not released and the antibacterial activity of the nanomaterial was severely decreased.

A different, yet somewhat similar use for TiO_2_ NPs was tested by Bakhshizadeh et al. [91]. They constructed a nanosystem comprised of titania core coated with mitoxantrone-loaded polymer based on diacrylated polycaprolactone as a biodegradable cross-linker and methacrylic acid or 4-vinylpyridine as the functional monomer. Authors utilized the ability of TiO_2_ NPs to emit visible light upon irradiation with X-rays—the so-called scintillation nanoparticles. It was established that light emission of the NPs matched the absorption spectrum of mitoxantrone. In this way, a simple but effective method for light delivery in PDT for more deeply lying tumors was developed. The nanoparticles were than tested for their cytotoxicity against HT1080 cells (fibrosarcoma). Cell viability was found to be unchanged when the NPs alone were tested, while around 70% of cells were viable after X-ray irradiation.

❖ **To sum up**

○ TiO_2_ in combination with anticancer agents offers a platform for more efficient delivery of chemotherapeutics. Thanks to either release mechanism used: pH-dependent, irradiation-triggered, or simple delivery, drug release in tumor cells is much higher than in healthy cells. As a result, the amount of drug used in the treatment can be significantly lower, while the pharmacological effect is maintained and fewer potential adverse effects occur. This can still be improved by not only combining different therapies, as shown by utilization of PDT and classical chemotherapy, but also by assessment of a mixture of anticancer drugs and other anticancer therapies.
○ Most research concerns the combination of TiO_2_ NPs with doxorubicin, and the results are encouraging.
○ The decreased cytotoxicity of doxorubicin-loaded on upconverting nanoparticles containing TiO_2_ was observed in many studies.
○ Mixed chemotherapy and PDT were studied after encapsulation of doxorubicin into “capsules” composed of Au-TiO_2_ NPs. The diffusion of the drug around the tumor was noted as the result of the acidic environment of the tissue core.

## 9. Other Applications of TiO_2_ Nanoparticles in Medicine

Applications of titanium dioxide in medicine are going further than the design of drug delivery systems or applications as vehicles for chemotherapeutics. Titanium dioxide NPs have been applied in pharmacy, especially in pharmaceutical chemistry and technology, as well as medicine, including growing areas related to dentistry and surgery. The selected TiO_2_ forms of potential applications in dentistry, surgery, and pharmacy discussed in this chapter are summarized in Table 3.

In dentistry, the photochemical activity of titanium dioxide was utilized for the improvement of tooth personal care and teeth whitening. This property was demonstrated by Cuppini et al., who studied TiO_2_ gel bearing H_2_O_2_ and methylene blue [97]. They noticed that the combination of TiO_2_ with H_2_O_2_ allows for reducing the time necessary for tooth bleaching by 30 min of gel-tooth direct contact significantly [97]. Importantly, Kurzmann et al. reported initial toxicity studies of TiO_2_ based gels for tooth bleaching. They observed that diluted gel formulations tested against L-929 cells, 3T3 cells, and gingival fibroblasts did not reveal any noticeable reduction in cell viability. The authors suggested additional experiments aiming to assess the TiO_2_ gel toxicity [92]. Sodagar et al. solved the problem of caries next to brackets in orthodontic treatment. They proposed the addition of TiO_2_ NPs at the concentration up to 10% into the orthodontic bond. The presence of TiO_2_ in the composite decreased the colony counts of *Streptococcus mutans* and *S. sanguinis*. Unfortunately, the presence of titanium dioxide NPs resulted also in loss in the shear bond strength in comparison to unmodified composite. Finally, the addition of up to 5% of TiO_2_ helped to achieve a compromise between bacterial growth reduction and loss in the shear bonding strength [98]. During the orthodontic treatment with a fixed appliance, the caries lesions are commonly spotted, especially next to orthodontic brackets. It is a result of difficult access to these areas in daily personal care. Therefore, the development of a new class of orthodontic bonds with bactericidal activity is very desirable. Sharma et al. reported the necessary shear bond strength for orthodontic treatment as 5.9–8 MPa [99]. The TiO_2_ modified composite developed by Sodagar et al. with shear bond strength 13.9 MPa seems to be a promising material. It should be analyzed whether it is suitable for safe debonding of orthodontic brackets. In another study, Sun et al. prepared TiO_2_ nanotubes and loaded them with tetracycline [100]. They carried out several tests against *Porphyromonas gingivalis.* Pure TiO_2_ and loaded nanotubes showed great adhesion potential and antibacterial properties. Also, tetracycline was released quickly within 15 min of the experiment and the material was stabilized in 90 min [100]. TiO_2_ nanoparticles have not only antibacterial but also antifungal properties. Huang et al. prepared Co-Cr alloy, on which they deposited a thin layer of TiO_2_. Several tests under UV-irradiation revealed that the prepared material exhibited significant antifungal activity and that it can be considered in the future against denture stomatitis [101].

Another significant area of titanium dioxide application is tooth hypersensitivity treatment, which is described as “sharp pain arising from exposed dentine in response to stimuli”. Hypersensitivity is stimulated by warm, tactile, chemicals, osmotic cause, and others. It is estimated that 15% of the population suffers from tooth hypersensitivity. The majority of them are at the age between 20 and 40. Dentin is the main tooth structure that provides a skeleton, which is covered with a hard structure—an enamel. In dentine, there are located dentinal tubules, small channels through which the dentinal fluid is flowing from pulp to outside. In an insensitive tooth, only a few channels are opened. Due to the abrasion processes and gingival recession, more and more dentinal tubules are exposed to external factors. A hydrodynamic mechanism of tooth hypersensitivity is considered as the most accurate. According to this mechanism, mentioned factors cause an increase in the outward flow of dentine fluid. This phenomenon is a reason for the growth pressure, which directly triggers a mechanoreceptor response [102]. Commercial toothpaste containing TiO_2_ is available on the market. Despite this fact, there is still interest in a new form of TiO_2_ in order to achieve improved or better-occluding properties. For example, Onwubu et al. prepared eggshell-TiO_2_ composite for occluding opened dentine tubules [93]. The authors noticed that the developed nanomaterial provided efficient dentine occlusion and allowed the covering of a large area of dentine. They compared their dentine occluding capability with commercially available toothpaste. Moreover, it was proved that presented eggshell-TiO_2_ was resistant to acidic conditions. This property is highly relevant because of a decreased pH in the oral cavity after sugar consumption [93]. In another study, Sereda et al. functionalized titanium dioxide with chondroitin sulfate to increase the dentine adhesiveness property of nanomaterial. This TiO_2_-based nanomaterial deposited on dentine hampered the adhesion of acidogenic bacteria [103], which play a crucial role in caries formation.

Titanium dioxide scaffolds have been considered as interesting materials for medical applications, including the preparation of implants for surgery in bone tissue engineering [104]. Coating the surface of titanium endoprostheses with a bioactive titanium dioxide layer was also studied in terms of the efficiency of fibrointegration [94]. Atomic layer deposition coating of TiO_2_ nano-thin films on magnesium-zinc alloys was found to enhance cytocompatibility for bioresorbable vascular stents [105].

In pharmaceutical sciences, titanium dioxide has been used as a pharmaceutical excipient in the manufacture of tablets as well as catalytic systems able to eliminate dangerous chemical and pharmaceutical pollutants. Lately, Hautala et al. have developed an ultrathin coating of minitablets by atomic layer deposition [106]. The obtained coat provides the tablets with improved properties related to the accelerated disintegration in the *in vitro* study [106]. TiO_2_ nanoparticles can also be used for masking the bitter taste of drugs, which was presented by Amin et al. when the addition of TiO_2_ NPs to azithromycin resulted in an improvement of taste and prolonged physicochemical stability of particles for 90 days [107]. Undoubtedly, a very evolving research field related to the tremendous amount of pharmaceutical pollutants in the environment is related to the development of photocatalytic systems based on TiO_2_ NPs aiming to degrade and eliminate them from aquatic systems. The fast and efficient degradation of phenol using TiO_2_ NPs was studied by Zulfiqar et al. [95]. The authors designed a photocatalytic system based on TiO_2_, which was applied for efficient removal of phenol with 99.48% yield during 540 min irradiation time [95]. Rendel et al. [108] studied the photodegradation kinetics of caffeine under different UV C doses at 254 nm in the presence of hydrogen peroxide and TiO_2_ nanopowder. The removal rate of caffeine was higher than 95% for both agents separately [108]. In another study, photocatalytic degradation of atenolol by TiO_2_ irradiated with an ultraviolet light-emitting diode was performed, and included a catalyst crystal phase (anatase TiO_2_, rutile TiO_2_, and mixed-phase), catalyst dosage, in the presence of co-existing anions, cations, and pH [96]. Recent approaches towards light-assisted photocatalytic removal of aqueous pharmaceutical pollutants have demonstrated the utility of titania and its derivatives not only with UV, but also with visible light, which was recently reviewed by Majumdar and Pal [109]. Moreover, photocatalytic degradation of pharmaceuticals, such as carbamazepine, diclofenac, and sulfamethoxazole by semiconductor (including titanium dioxide) and carbon materials, was also the subject of interesting research performed by Mestre and Carvalho [110]. In addition, TiO_2_-coated glass slides were applied for the study of a variety of oxidation reactions, including drug candidates and their oxidation products [111]. There are also reports showing that TiO_2_ NPs revealed attractive potential as photocatalysts for anti-inflammatory, analgesic drugs [112], cyanide [113], atenolol [114], carbamazepine [115], *β*-blockers [116], and betamethasone-17 valerate [117]. Moreover, Ruokolainen et al. performed oxidation of tyrosine-phosphopeptides with TiO_2_ particles, suggesting their potential as photocatalysts for biomolecules [118].

❖ **To sum up**

○ TiO_2_ NPs were evaluated for use in pharmacy, especially in pharmaceutical chemistry and technology, as well as medicine, including growing areas related to dentistry and surgery.
○ In dentistry, the photochemical activity of TiO_2_ was utilized for the improvement of tooth personal care and teeth whitening.
○ Eggshell-TiO_2_ composite was found useful for occluding opened dentine tubules, allowing for efficient dentine occlusion.
○ TiO_2_ scaffolds were applied for the preparation of implants for surgery in bone tissue engineering.
○ In pharmaceutical sciences, TiO_2_ was applied as a pharmaceutical excipient in the manufacture of tablets, as well as a catalytic system able to eliminate dangerous chemical and pharmaceutical pollutants

## 10. Summary

Recently, many studies have been focused on the broad applications of titania in technology and medicine, from dye-sensitized solar cells, photodynamic therapy, to water remediation. It is possible because these nanoparticles reveal excellent photochemical properties and high biocompatibility. Moreover, TiO_2_ particles are quite cheap and accessible components. Photosensitizing properties and manufacturing costs of these materials are usually related to the number of active sites on their surface. After irradiation, they can produce reactive oxygen species that spread from the nanoparticles and induce cell death in the neighboring tissues. Therefore, applications of TiO_2_ nano- and microparticles in photodynamic therapy are widely explored. Another application of TiO_2_ nanoparticles is related to their use as a drug carrier, allowing drugs to reach diseased areas of the body while keeping healthy tissues unharmed. Therefore, much attention must be paid to develop novel formulations allowing to direct the active substances to target cells and minimize the side effects. A plethora of various studies above indicate that an increase of TiO_2_ therapeutic efficiency can be reached using targeted drug delivery systems and nanocomposites. It is possible because the surface of TiO_2_ NPs can also be labeled with antibodies or markers in order to design drug delivery towards selected, diseased areas.

For more extensive usage of TiO_2_ NPs, some of their drawbacks should be overcome. The applications of neat TiO_2_ nanoparticles in photodynamic therapy are limited by the necessity to use UV light for their excitation. Fortunately, numerous methods of doping and functionalization of the TiO_2_ NPs have been developed. These modifications result in the shift of NPs absorption band towards longer wavelengths, desirable for PDT. Combinations of titania with other nanoparticles such as up-conversion nanoparticles also enable it to bypass the direct use of UV light. Titanium dioxide particles are prone to form agglomerates in physiological pH, hampering their solubility. To prevent this unwelcome issue, the functionalization of TiO_2_ with bulky substituents like PEG chains or the use of surfactants was proposed. It could also enable the uniform distribution of the nanoparticles and thus repeatability of dosing accompanied by clinical effect. In the end, the crucial issue concerning the use of TiO_2_ in the medical field has to be pointed out. Available data do not present a full profile of the TiO_2_ particles’ fate in the body as well as their toxicity. Therefore, extensive studies in this area are urgently needed.

The studies discussed in this review indicate that both composites and conjugates of titania with various molecules and biomolecules can significantly improve their broader applications in medicine.

## Figures and Tables

**Table 1:** Synthesis, physicochemical characteristics, and medical applications of selected TiO_2_ nanoparticles (NPs) combined with photosensitizers.

Ref.	Shape of NPs (Characteristics)	Photosensitizer	Method of Synthesis	Medical/Biological Use
[57]	P25 TiO_2_ (75% anatase and 25% rutile, size 25 nm)	5,10,15,20-tetrakis(2,6-difluorosulfonylophenyl)porphyrin and its zinc(II) complex	commercial distribution	PACT against *S. aureus*, *E. coli*
[58]	N-TiO_2_-NH_2_ (size: 20–30 nm)	Aluminum(III) phthalocyanine chloride tetrasulfonate	N-doping by calcination of commercially available anatase TiO_2_ NPs in ammonia atmosphere	PDT against cancer (HeLa and KB cell lines)
[59]	N-TiO_2_-NH_2_ (size: 20–30 nm)	Aluminum(III) phthalocyanine chloride tetrasulfonate	N-doping by calcination of commercially available anatase TiO_2_ NPs in ammonia atmosphere	PDT against cancer (HeLa cell line)
[60]	anatase (size: 23 nm spheres)	subphthalocyanine derivatives	from TiCl_4_ and benzyl alcohol; macrocycle deposition overnight in THF	PDT against breast and cervical tumors
[61]	anatase (23 nm spheres)	Zinc(II) phthalocyanine derivatives	from TiCl_4_ and benzyl alcohol; macrocycle deposition overnight in THF	PACT against: *S. aureus*
[62]	anatase (23 nm spheres)	Subphthalocyanine derivative	from TiCl_4_ and benzyl alcohol; macrocycle deposition overnight in THF	PACT against *S. aureus*, *E. coli*
[63]	anatase (size—25 nm)	Zinc(II) tetrakis(3-dodecylpyridyloxy)phthalocyanine (mixture of isomers)	deposition in pyridine/ethanol mixture	PACT against MRSA, *Salmonella enteritidis*
[64]	no data presented	Zinc(II) phthalocyanine	sol-gel method	PACT against *Leishmania chagasi, Leishmania panamensis*; PDT against human liver cancer cell line
[65]	anatase/rutile film (600 nm in film thickness, 100 nm grain size)	Copper tetracarboxyphthalocyanines (mixture of isomers)	anodization	PACT against MRSA
[66]	TiO_2_ nanowhiskers (size < 100 nm)	tetrasulphonatophenyl porphyrin	undefined deposition in water	PDT and bioimaging of rheumatoid arthritis
[67]	TiO_2_ nanowhiskers	tetrasulphonatophenyl porphyrin	undefined; deposition in water	PDT of diabetes mellitus
[68]	P25 TiO_2_ (75% anatase and 25% rutile, size—21 nm)	Chlorin e6	silylation with or without PEGylation	PDT against glioblastoma cell
[69]	no data (size—100 nm)	methylene blue used in mixture but without grafting the NPs	commercial distribution	PACT against: *S. aureus*, *E. coli*, and *Candida albicans*

THF—tetrahydrofuran; NPs—nanoparticles.

**Table 2:** Synthesis, physicochemical characteristics, and medical applications of selected TiO_2_ NPs in combination with doxorubicin.

Ref.	Shape of Nanoparticles (Characteristics)	Method of Synthesis	Medical/Biological Use
[83]	ZnPc@TiO_2__CHCl_3_ (20 nm) ZnPc@TiO_2__THF (125 nm) ZnPc@TiO_2__CHCl_3_/THF (13 nm); mostly anatase with small addition of rutile	NPs—commercially; nanotubes—from titanium(IV) isopropoxide in a sol-gel method followed by hydrothermal treatment; deposition of ZnPc in CHCl_3_, THF or 1:1 *v/v* CHCl_3_/THF	PDT, bioimaging and doxorubicin delivery (tested on HeLa cells)
[84]	UCNPs@SiO_2_@TiO_2_ (TiO_2_ shell thickness—5–6 nm)	TiO_2_ was grown on UCNPs@SiO_2_-NH_2_ NPs from titanium diisopropoxide bis(acetylacetonate); further hydrothermal treatment yielded crystalline structure	PDT in cancer treatment mixed with doxorubicin (tested on HeLa cells)
[85]	diamond-shaped mesoporous TiO_2_ (220 nm in width, 250 nm in length, 40 nm thick, pore size—4.1 nm)	from Ti(IV) isopropoxide at 28 °C, followed by silylation and PEGylation	pH-responsive drug delivery vehicles for cancer therapy
[86]	TiO_2_ nanowhiskers (width 80 nm, length range—200–5000 nm)	K_2_CO_3_ with TiO_2_ heated at 810 °C, soaked in distilled water for about 7 days, dried, and calcinated	PDT with daunorubicin delivery against hepatocarcinoma cells
[87]	0.3 µm TiO_2_ nanotube array (single nanotube diameter—90 nm)	growth of TiO_2_ nanotubes in a glycerol/water/NH_4_F mixture, then annealing to form anatase	Visible-light-triggered release of ampicillin
[88]	NaYF_4_:Yb/Tm-TiO_2_ (sphere-shaped) (20–40 nm)	TiO_2_ NPs prepared by solvothermal method from tetrabutyl titanate; trifluoroacetates of lantanides were mixed with TiO_2_ NPs and thermally treated; further functionalization included PEGylation, silylation and conjugation of folic acid	PDT with doxorubicin delivery tested on drug-resistant breast cancers
[89]	UCNPs@mSiO_2_/TiO_2_ (30 nm of silica/titania shell thickness)	silica coating was synthesized on UCNPs with tetraethylorthosilicate, silylated and reacted with tetrabutyl titanate followed by calcination to yield anatase phase	PDT mixed with doxorubicin delivery against HeLa cells)
[90]	TiO_2_ (anatase, 10 nm) Au-TiO_2_ (1–30 nm)	TiO_2_ from butyl titanate by solvothermal method; Au-TiO_2_ by solvothermal method using mixture of butyl titanate and HAuCl_4_; both were followed by calcination.	PDT and doxorubicin delivery tested on breast cancer cells

**Table 3:** Synthesis, physicochemical characteristics and applications of selected TiO_2_ NPs in dentistry, surgery and pharmacy.

Ref.	Shape of Nanoparticles (Characteristics)	Method of Synthesis	Medical/Biological Use
[92]	TiO_2_ (anatase, 25 nm) TiO_2_/Ag NPs	commercial distribution of TiO_2_ anatase powder was mixed with silver nitrate, reduced and heated at 300 °C	toxicity reduction of teeth whitening gels
[93]	TiO_2_ (anatase, ≤15 µm) Eggshell-TiO_2_ composite (irregular, spherical shape particles, ≤13 nm)	undefined/commercial distribution of TiO_2_, eggshell powder with TiO_2_ was ground in ball mill	occluding opened dentine tubules
[94]	TiO_2_ (anatase, 10 nm)	undefined/commercial distribution	improving of endoprotheses biocompatibility
[95]	P25 (anatase/rutile 8:2, 21 nm)	commercial distribution	photocatalytic degradation of phenol
[96]	TiO_2_ (anatase, 20–50 nm) TiO_2_ (rutile, 50–100 nm) TiO_2_ mixed phase (anatase/rutile 83:17, 20–50 nm)	commercial distribution	photocatalytic degradation of atenolol

## References

[1] Horikoshi S., Serpone N. (2013). Introduction to nanoparticles. Microw. Nanopart. Synth. Fundam. Appl..

[2] Youssef Z., Vanderesse R., Colombeau L., Baros F., Roques-Carmes T., Frochot C., Wahab H., Toufaily J., Hamieh T., Acherar S. (2017). The application of titanium dioxide, zinc oxide, fullerene, and graphene nanoparticles in photodynamic therapy. Cancer Nanotechnol..

[3] ISO/TS 80004-2:2015(en) Nanotechnologies—Vocabulary—Part 2: Nano-objects. https://www.iso.org/obp/ui/#iso:std:iso:ts:80004:-2:ed-1:v1:en.

[4] Caep O., Huisman C.L., Reller A. (2004). Photoinduced Reactivity of Titanium Dioxide. Prog. Solid State Chem..

[5] Matsunaga T., Tomoda R., Nakajima T., Wake H. (1985). Photoelectrochemical sterilization of microbial cells by semiconductor powders. FEMS Microbiol. Lett..

[6] Xu J., Sun Y., Huang J., Chen C., Liu G., Jiang Y., Zhao Y., Jiang Z. (2007). Photokilling cancer cells using highly cell-specific antibody–TiO_2_ bioconjugates and electroporation. Bioelectrochemistry.

[7] Ni W., Li M., Cui J., Xing Z., Li Z., Wu X., Song E., Gong M., Zhou W. (2017). 808 nm light triggered black TiO_2_ nanoparticles for killing of bladder cancer cells. Mater. Sci. Eng. C.

[8] Carlander U., Li D., Jolliet O., Emond C., Johanson G. (2016). Toward a general physiologically-based pharmacokinetic model for intravenously injected nanoparticles. Int. J. Nanomed..

[9] Lin Z., Monteiro-Riviere N.A., Riviere J.E. (2015). Pharmacokinetics of metallic nanoparticles: Pharmacokinetics of metallic nanoparticles. WIREs NanoMed. Nanobiotechnol..

[10] Janer G., Mas del Molino E., Fernández-Rosas E., Fernández A., Vázquez-Campos S. (2014). Cell uptake and oral absorption of titanium dioxide nanoparticles. Toxicol. Lett..

[11] Wang J., Zhou G., Chen C., Yu H., Wang T., Ma Y., Jia G., Gao Y., Li B., Sun J. (2007). Acute toxicity and biodistribution of different sized titanium dioxide particles in mice after oral administration. Toxicol. Lett..

[12] Bachler G., von Goetz N., Hungerbuhler K. (2015). Using physiologically based pharmacokinetic (PBPK) modeling for dietary risk assessment of titanium dioxide (TiO_2_) nanoparticles. Nanotoxicology.

[13] Fabian E., Landsiedel R., Ma-Hock L., Wiench K., Wohlleben W., van Ravenzwaay B. (2008). Tissue distribution and toxicity of intravenously administered titanium dioxide nanoparticles in rats. Arch. Toxicol..

[14] Geraets L., Oomen A.G., Krystek P., Jacobsen N.R., Wallin H., Laurentie M., Verharen H.W., Brandon E.F., de Jong W.H. (2014). Tissue distribution and elimination after oral and intravenous administration of different titanium dioxide nanoparticles in rats. Part. Fibre Toxicol..

[15] Xie G., Wang C., Sun J., Zhong G. (2011). Tissue distribution and excretion of intravenously administered titanium dioxide nanoparticles. Toxicol. Lett..

[16] Wu J., Liu W., Xue C., Zhou S., Lan F., Bi L., Xu H., Yang X., Zeng F.-D. (2009). Toxicity and penetration of TiO_2_ nanoparticles in hairless mice and porcine skin after subchronic dermal exposure. Toxicol. Lett..

[17] Crosera M., Prodi A., Mauro M., Pelin M., Florio C., Bellomo F., Adami G., Apostoli P., Palma G.D., Bovenzi M. (2015). Titanium Dioxide Nanoparticle Penetration into the Skin and Effects on HaCaT Cells. Int. J. Environ. Res. Public Health.

[18] Yin J.-J., Liu J., Ehrenshaft M., Roberts J.E., Fu P.P., Mason R.P., Zhao B. (2012). Phototoxicity of nano titanium dioxides in HaCaT keratinocytes—Generation of reactive oxygen species and cell damage. Toxicol. Appl. Pharmacol..

[19] Lee K.P., Trochimowicz H.J., Reinhardt C.F. (1985). Pulmonary response of rats exposed to titanium dioxide (TiO_2_) by inhalation for two years. Toxicol. Appl. Pharmacol..

[20] Vandebriel R.J., Vermeulen J.P., van Engelen L.B., de Jong B., Verhagen L.M., de la Fonteyne-Blankestijn L.J., Hoonakker M.E., de Jong W.H. (2018). The crystal structure of titanium dioxide nanoparticles influences immune activity in vitro and in vivo. Part. Fibre Toxicol..

[21] Ganguly D., Haak S., Sisirak V., Reizis B. (2013). The role of dendritic cells in autoimmunity. Nat. Rev. Immunol..

[22] Shacter E., Weitzman S.A. (2002). Chronic inflammation and cancer. Oncology.

[23] Madhubala V., Pugazhendhi A., Thirunavukarasu K. (2019). Cytotoxic and immunomodulatory effects of the low concentration of titanium dioxide nanoparticles (TiO_2_ NPs) on human cell lines—An in vitro study. Process Biochem..

[24] Rehman F.U., Zhao C., Jiang H., Selke M., Wang X. (2016). Protective effect of TiO_2_ nanowhiskers on Tetra Sulphonatophenyl Porphyrin (TSPP) complexes induced oxidative stress during photodynamic therapy. Photodiagnosis Photodyn. Ther..

[25] Gupta S.M., Tripathi M. (2011). A review of TiO_2_ nanoparticles. Chin. Sci. Bull..

[26] Noman M.T., Ashraf M.A., Ali A. (2019). Synthesis and applications of nano-TiO_2_: A review. Environ. Sci. Pollut. Res..

[27] Chen X., Mao S.S. (2007). Titanium Dioxide Nanomaterials: Synthesis, Properties, Modifications, and Applications. Chem. Rev..

[28] Muniandy S.S., Kaus N.H.M., Jiang Z.-T., Altarawneh M., Lee H.L. (2017). Green synthesis of mesoporous anatase TiO_2_ nanoparticles and their photocatalytic activities. RSC Adv..

[29] Falk G.S., Borlaf M., López-Muñoz M.J., Fariñas J.C., Rodrigues Neto J.B., Moreno R. (2018). Microwave-assisted synthesis of TiO_2_ nanoparticles: Photocatalytic activity of powders and thin films. J. Nanopart. Res..

[30] Macyk W., Szaciłowski K., Stochel G., Buchalska M., Kuncewicz J., Łabuz P. (2010). Titanium (IV) complexes as direct TiO_2_ photosensitizers. Coord. Chem. Rev..

[31] Yuan R., Zhou B., Hua D., Shi C., Ma L. (2014). Effect of metal-ion doping on the characteristics and photocatalytic activity of TiO_2_ nanotubes for the removal of toluene from water. Water Sci. Technol..

[32] Gupta N., Pal B. (2013). Photocatalytic activity of transition metal and metal ions impregnated TiO_2_ nanostructures for iodide oxidation to iodine formation. J. Mol. Catal. A Chem..

[33] Savinkina E., Obolenskaya L., Kuzmicheva G. (2015). Efficiency of sensitizing nano-titania with organic dyes and peroxo complexes. Appl. Nanosci..

[34] Kondratyeva I., Orzeł Ł., Kobasa I., Doroshenko A., Macyk W. (2016). Photosensitization of titanium dioxide with 4′-dimethylaminoflavonol. Mater. Sci. Semicond. Process..

[35] Rochkind M., Pasternak S., Paz Y. (2014). Using Dyes for Evaluating Photocatalytic Properties: A Critical Review. Molecules.

[36] Feng X., Zhang S., Wu H., Lou X. (2015). A novel folic acid-conjugated TiO_2_-SiO_2_ photosensitizer for cancer targeting in photodynamic therapy. Colloids Surf. B Biointerfaces.

[37] Zaleska A. (2008). Doped-TiO_2_: A Review. Recent Pat. Eng..

[38] Guiot C., Spalla O. (2013). Stabilization of TiO_2_ Nanoparticles in Complex Medium through a pH Adjustment Protocol. Environ. Sci. Technol..

[39] Xu F. (2018). Review of analytical studies on TiO_2_ nanoparticles and particle aggregation, coagulation, flocculation, sedimentation, stabilization. Chemosphere.

[40] Kubiak A., Siwińska-Ciesielczyk K., Goscianska J., Dobrowolska A., Gabała E., Czaczyk K., Jesionowski T. (2019). Hydrothermal-assisted synthesis of highly crystalline titania-copper oxide binary systems with enhanced antibacterial properties. Mater. Sci. Eng. C.

[41] Lagopati N., Kitsiou P.V., Kontos A.I., Venieratos P., Kotsopoulou E., Kontos A.G., Dionysiou D.D., Pispas S., Tsilibary E.C., Falaras P. (2010). Photo-induced treatment of breast epithelial cancer cells using nanostructured titanium dioxide solution. J. Photochem. Photobiol. A Chem..

[42] Wang C., Cao S., Tie X., Qiu B., Wu A., Zheng Z. (2011). Induction of cytotoxicity by photoexcitation of TiO_2_ can prolong survival in glioma-bearing mice. Mol. Biol. Rep..

[43] Feng X., Zhang S., Lou X. (2013). Controlling silica coating thickness on TiO_2_ nanoparticles for effective photodynamic therapy. Colloids Surf. B Biointerfaces.

[44] Shanmugapriya K., Kang H.W. (2019). Engineering pharmaceutical nanocarriers for photodynamic therapy on wound healing: Review. Mater. Sci. Eng. C.

[45] Archana D., Singh B.K., Dutta J., Dutta P.K. (2013). In vivo evaluation of chitosan–PVP–titanium dioxide nanocomposite as wound dressing material. Carbohydr. Polym..

[46] Li G., Lv L., Fan H., Ma J., Li Y., Wan Y., Zhao X.S. (2010). Effect of the agglomeration of TiO_2_ nanoparticles on their photocatalytic performance in the aqueous phase. J. Colloid Interface Sci..

[47] Kayani Z.N., Riaz S., Naseem S. (2020). Magnetic and antibacterial studies of sol-gel dip coated Ce doped TiO_2_ thin films: Influence of Ce contents. Ceram. Int..

[48] Shah Z., Nazir S., Mazhar K., Abbasi R., Samokhvalov I.M. (2019). PEGylated doped- and undoped-TiO_2_ nanoparticles for photodynamic Therapy of cancers. Photodiagn. Photodyn. Ther..

[49] Zeni P.F., Santos D.P.D., Canevarolo R.R., Yunes J.A., Padilha F.F., de Albuquerque J.R.L.C., Egues S.M., Hernández-Macedo M.L. (2018). Photocatalytic and Cytotoxic Effects of Nitrogen-Doped TiO_2_ Nanoparticles on Melanoma Cells. J. Nanosci. Nanotechnol..

[50] Shang H., Han D., Ma M., Li S., Xue W., Zhang A. (2017). Enhancement of the photokilling effect of TiO_2_ in photodynamic therapy by conjugating with reduced graphene oxide and its mechanism exploration. J. Photochem. Photobiol. B Biol..

[51] Ismail A.F.M., Ali M.M., Ismail L.F.M. (2014). Photodynamic therapy mediated antiproliferative activity of some metal-doped ZnO nanoparticles in human liver adenocarcinoma HepG2 cells under UV irradiation. J. Photochem. Photobiol. B Biol..

[52] Ghaderi S., Ramesh B., Seifalian A.M. (2011). Fluorescence nanoparticles “quantum dots” as drug delivery system and their toxicity: A review. J. Drug Target..

[53] Jia X., Jia L. (2012). Nanoparticles Improve Biological Functions of Phthalocyanine Photosensitizers Used for Photodynamic Therapy. Curr. Drug Metab..

[54] Di Carlo G., Biroli A.O., Tessore F., Caramori S., Pizzotti M. (2018). β-Substituted ZnII porphyrins as dyes for DSSC: A possible approach to photovoltaic windows. Coord. Chem. Rev..

[55] Zhang L., Cole J.M. (2015). Anchoring Groups for Dye-Sensitized Solar Cells. ACS Appl. Mater. Interfaces.

[56] Rehman F.U., Zhao C., Jiang H., Wang X. (2016). Biomedical applications of nano-titania in theranostics and photodynamic therapy. Biomater. Sci..

[57] Sułek A., Pucelik B., Kuncewicz J., Dubin G., Dąbrowski J.M. (2019). Sensitization of TiO_2_ by halogenated porphyrin derivatives for visible light biomedical and environmental photocatalysis. Catal. Today.

[58] Pan X., Xie J., Li Z., Chen M., Wang M., Wang P.-N., Chen L., Mi L. (2015). Enhancement of the photokilling effect of aluminum phthalocyanine in photodynamic therapy by conjugating with nitrogen-doped TiO_2_ nanoparticles. Colloids Surf. B Biointerfaces.

[59] Pan X., Liang X., Yao L., Wang X., Jing Y., Ma J., Fei Y., Chen L., Mi L. (2017). Study of the Photodynamic Activity of N-Doped TiO_2_ Nanoparticles Conjugated with Aluminum Phthalocyanine. Nanomaterials.

[60] Yurt F., Ocakoglu K., Ince M., Colak S.G., Er O., Soylu H.M., Gunduz C., Biray Avci C., Caliskan Kurt C. (2018). Photodynamic therapy and nuclear imaging activities of zinc phthalocyanine-integrated TiO_2_ nanoparticles in breast and cervical tumors. Chem. Biol. Drug Des..

[61] Tunçel A., Öztürk İ., Ince M., Ocakoglu K., Hoşgör-Limoncu M., Yurt F. (2019). Antimicrobial photodynamic therapy against *Staphylococcus aureus* using zinc phthalocyanine and zinc phthalocyanine-integrated TiO_2_ nanoparticles. J. Porphyr. Phthalocyanines.

[62] Ozturk I., Tunçel A., Ince M., Ocakoglu K., Hoşgör-Limoncu M., Yurt F. (2018). Antibacterial properties of subphthalocyanine and subphthalocyanine-TiO_2_ nanoparticles on *Staphylococcus aureus* and *Escherichia coli*. J. Porphyr. Phthalocyanines.

[63] Mantareva V., Eneva I., Kussovski V., Borisova E., Angelov I. Antimicrobial photodisinfection with Zn(II) phthalocyanine adsorbed on TiO_2_ upon UVA and red irradiation. Proceedings of the 18th International School on Quantum Electronics: Laser Physics and Applications; International Society for Optics and Photonics.

[64] Lopez T., Ortiz E., Alvarez M., Navarrete J., Odriozola J.A., Martinez-Ortega F., Páez-Mozo E.A., Escobar P., Espinoza K.A., Rivero I.A. (2010). Study of the stabilization of zinc phthalocyanine in sol-gel TiO_2_ for photodynamic therapy applications. NanoMed. Nanotechnol. Biol. Med..

[65] Perillo P.M., Getz F.C. (2016). Dye Sensitized TiO_2_ Nanopore Thin Films with Antimicrobial Activity Against Methicillin Resistant Staphylococcus Aureus Under Visible Light. World J. Appl. Chem..

[66] Zhao C., Rehman F.U., Yang Y., Li X., Zhang D., Jiang H., Selke M., Wang X., Liu C. (2015). Bio-imaging and Photodynamic Therapy with Tetra Sulphonatophenyl Porphyrin (TSPP)-TiO_2_ Nanowhiskers: New Approaches in Rheumatoid Arthritis Theranostics. Sci. Rep..

[67] Rehman F., Zhao C., Jiang H., Selke M., Wang X.D. (2016). Photoactivated TiO_2_ Nanowhiskers and Tetra Sulphonatophenyl Porphyrin Normoglycemic Effect on Diabetes Mellitus During Photodynamic Therapy. J. Nanosci. Nanotechnol..

[68] Youssef Z., Jouan-Hureaux V., Colombeau L., Arnoux P., Moussaron A., Baros F., Toufaily J., Hamieh T., Roques-Carmes T., Frochot C. (2018). Titania and silica nanoparticles coupled to Chlorin e6 for anti-cancer photodynamic therapy. Photodiagnosis Photodyn. Ther..

[69] Tuchina E.S., Tuchin V.V. (2010). TiO_2_ nanoparticle enhanced photodynamic inhibition of pathogens. Laser Phys. Lett..

[70] Yordanova A., Eppard E., Kürpig S., Bundschuh R.A., Schönberger S., Gonzalez-Carmona M., Feldmann G., Ahmadzadehfar H., Essler M. (2017). Theranostics in nuclear medicine practice. Onco Targets Ther..

[71] Makhseed S., Machacek M., Alfadly W., Tuhl A., Vinodh M., Novakova V., Kubat P., Rudolf E., Zimcik P. (2013). Water-soluble non-aggregating zinc phthalocyanine and in vitro study for photodynamic therapy. Chem. Commun..

[72] Yurt F., Ince M., Colak S.G., Ocakoglu K., Er O., Soylu H.M., Gunduz C., Avci C.B., Kurt C.C. (2017). Investigation of in vitro PDT activities of zinc phthalocyanine immobilised TiO_2_ nanoparticles. Int. J. Pharm..

[73] Erdural B.K., Yurum A., Bakir U., Karakas G. (2008). Antimicrobial properties of titanium nanoparticles. Functionalized Nanoscale Materials, Devices and Systems.

[74] Shirai R., Miura T., Yoshida A., Yoshino F., Ito T., Yoshinari M., Yajima Y. (2016). Antimicrobial effect of titanium dioxide after ultraviolet irradiation against periodontal pathogen. Dent. Mater. J..

[75] Itabashi T., Narita K., Ono A., Wada K., Tanaka T., Kumagai G., Yamauchi R., Nakane A., Ishibashi Y. (2017). Bactericidal and antimicrobial effects of pure titanium and titanium alloy treated with short-term, low-energy UV irradiation. Bone Jt. Res..

[76] Kou J., Dou D., Yang L. (2017). Porphyrin photosensitizers in photodynamic therapy and its applications. Oncotarget.

[77] Firestein G.S. (2003). Evolving concepts of rheumatoid arthritis. Nature.

[78] Marin J.J., Romero M.R., Blazquez A.G., Herraez E., Keck E., Briz O. (2009). Importance and limitations of chemotherapy among the available treatments for gastrointestinal tumours. Anti-Cancer Agents Med. Chem. (Former. Curr. Med. Chem.-Anti-Cancer Agents).

[79] Zimmermann S., Dziadziuszko R., Peters S. (2014). Indications and limitations of chemotherapy and targeted agents in non-small cell lung cancer brain metastases. Cancer Treat. Rev..

[80] Rivankar S. (2014). An overview of doxorubicin formulations in cancer therapy. J. Cancer Res. Ther..

[81] Lai Y.-K., Wang Q., Huang J.-Y., Li H.-Q., Chen Z., Zhao A.Z.-J., Wang Y., Zhang K.-Q., Sun H.-T., Al-Deyab S.S. (2016). TiO_2_ nanotube platforms for smart drug delivery: A review. Int. J. NanoMed..

[82] Raja G., Cao S., Kim D.-H., Kim T.-J. (2020). Mechanoregulation of titanium dioxide nanoparticles in cancer therapy. Mater. Sci. Eng. C.

[83] Flak D., Yate L., Nowaczyk G., Jurga S. (2017). Hybrid ZnPc@TiO_2_ nanostructures for targeted photodynamic therapy, bioimaging and doxorubicin delivery. Mater. Sci. Eng. C.

[84] Chen Y., Lin H., Tong R., An N., Qu F. (2017). Near-infrared light-mediated DOX-UCNPs@mHTiO_2_ nanocomposite for chemo/photodynamic therapy and imaging. Colloids Surf. B Biointerfaces.

[85] Wang Y., Wang Q., Zhang C. (2019). Synthesis of Diamond-Shaped Mesoporous Titania Nanobricks as pH-Responsive Drug Delivery Vehicles for Cancer Therapy. ChemistrySelect.

[86] Li Q., Wang X., Lu X., Tian H., Jiang H., Lv G., Guo D., Wu C., Chen B. (2009). The incorporation of daunorubicin in cancer cells through the use of titanium dioxide whiskers. Biomaterials.

[87] Xu J., Zhou X., Gao Z., Song Y.-Y., Schmuki P. (2016). Visible-Light-Triggered Drug Release from TiO_2_ Nanotube Arrays: A Controllable Antibacterial Platform. Angew. Chem. Int. Ed..

[88] Zeng L., Pan Y., Tian Y., Wang X., Ren W., Wang S., Lu G., Wu A. (2015). Doxorubicin-loaded NaYF4:Yb/Tm-TiO_2_ inorganic photosensitizers for NIR-triggered photodynamic therapy and enhanced chemotherapy in drug-resistant breast cancers. Biomaterials.

[89] Tong R., Lin H., Chen Y., An N., Wang G., Pan X., Qu F. (2017). Near-infrared mediated chemo/photodynamic synergistic therapy with DOX-UCNPs@mSiO_2_/TiO_2_-TC nanocomposite. Mater. Sci. Eng. C.

[90] Akram M.W., Raziq F., Fakhar-e-Alam M., Aziz M.H., Alimgeer K.S., Atif M., Amir M., Hanif A., Aslam Farooq W. (2019). Tailoring of Au-TiO_2_ nanoparticles conjugated with doxorubicin for their synergistic response and photodynamic therapy applications. J. Photochem. Photobiol. A Chem..

[91] Bakhshizadeh M., Sazgarnia A., Seifi M., Hadizadeh F., Rajabzadeh G., Mohajeri S.A. (2017). TiO_2_-based Mitoxantrone Imprinted Poly (Methacrylic acid-co-polycaprolctone diacrylate) Nanoparticles as a Drug Delivery System. Curr. Pharm. Des..

[92] Kurzmann C., Verheyen J., Coto M., Kumar R.V., Divitini G., Shokoohi-Tabrizi H.A., Verheyen P., De Moor R.J.G., Moritz A., Agis H. (2019). In vitro evaluation of experimental light activated gels for tooth bleaching. Photochem. Photobiol. Sci..

[93] Onwubu S.C., Mdluli P.S., Singh S., Tlapana T. (2019). A novel application of nano eggshell/titanium dioxide composite on occluding dentine tubules: An in vitro study. Braz. Oral Res..

[94] Shaikhaliyev A.I., Polisan A.A., Ivanov S.Y., Parkhomenko Y.N., Malinkovich M.D., Yarygin K.N., Arazashvili L.D. (2019). Effect of the Surface of Medical Titanium Endoprostheses on the Efficiency of Fibrointegration. J. Synch. Investig..

[95] Zulfiqar M., Samsudin M.F.R., Sufian S. (2019). Modelling and optimization of photocatalytic degradation of phenol via TiO_2_ nanoparticles: An insight into response surface methodology and artificial neural network. J. Photochem. Photobiol. A Chem..

[96] Ran Z., Wang L., Fang Y., Ma C., Li S. (2019). Photocatalytic Degradation of Atenolol by TiO_2_ Irradiated with an Ultraviolet Light Emitting Diode: Performance, Kinetics, and Mechanism Insights. Catalysts.

[97] Cuppini M., Leitune V.C.B., de Souza M., Alves A.K., Samuel S.M.W., Collares F.M. (2019). In vitro evaluation of visible light-activated titanium dioxide photocatalysis for in-office dental bleaching. Dent. Mater. J..

[98] Sodagar A., Akhoundi M.S.A., Bahador A., Jalali Y.F., Behzadi Z., Elhaminejad F., Mirhashemi A.H. (2017). Effect of TiO_2_ nanoparticles incorporation on antibacterial properties and shear bond strength of dental composite used in Orthodontics. Dent. Press J. Orthod..

[99] Sharma S., Singh G., Singh A., Tandon P., Nagar A. (2014). A comparison of shear bond strength of orthodontic brackets bonded with four different orthodontic adhesives. J. Orthod. Sci..

[100] Sun L., Xu J., Sun Z., Zheng F., Liu C., Wang C., Hu X., Xia L., Liu Z., Xia R. (2018). Decreased Porphyromonas gingivalis adhesion and improved biocompatibility on tetracycline-loaded TiO_2_ & nbsp;nanotubes: An in vitro study. Int. J. NanoMed..

[101] Huang L., Jing S., Zhuo O., Meng X., Wang X. (2017). Surface Hydrophilicity and Antifungal Properties of TiO_2_ Films Coated on a Co-Cr Substrate. BioMed Res. Int..

[102] Gillam D.G. (2015). Dentine Hypersensitivity: Advances in Diagnosis, Management, and Treatment.

[103] Sereda G., Rashwan K., Karels B., Fritza A. (2016). Novel Materials for Desensitizing and Remineralizing Dentifrices. Advanced Materials: TechConnect Briefs.

[104] Cuervo-Osorio G., Jiménez-Valencia A.M., Mosquera-Agualimpia C., Escobar-Sierra D.M. (2018). Manufacture of titanium dioxide scaffolds for medical applications. Revista Facultad de Ingeniería.

[105] Yang F., Chang R., Webster T. (2019). Atomic Layer Deposition Coating of TiO_2_ Nano-Thin Films on Magnesium-Zinc Alloys to Enhance Cytocompatibility for Bioresorbable Vascular Stents. Int. J. NanoMed..

[106] Hautala J., Kääriäinen T., Hoppu P., Kemell M., Heinämäki J., Cameron D., George S., Juppo A.M. (2017). Atomic layer deposition—A novel method for the ultrathin coating of minitablets. Int. J. Pharm..

[107] Amin F., Khan S., Shah S.M.H., Rahim H., Hussain Z., Sohail M., Ullah R., Alsaid M.S., Shahat A.A. (2018). A new strategy for taste masking of azithromycin antibiotic: Development, characterization, and evaluation of azithromycin titanium nanohybrid for masking of bitter taste using physisorption and panel testing studies. Drug Des. Dev. Ther..

[108] Rendel P.M., Rytwo G. (2020). Degradation kinetics of caffeine in water by UV/H_2_O_2_ and UV/TiO_2_. Desalin. Water Treat..

[109] Majumdar A., Pal A. (2019). Recent advancements in visible-light-assisted photocatalytic removal of aqueous pharmaceutical pollutants. Clean Technol. Environ. Policy.

[110] Mestre A.S., Carvalho A.P. (2019). Photocatalytic Degradation of Pharmaceuticals Carbamazepine, Diclofenac, and Sulfamethoxazole by Semiconductor and Carbon Materials: A Review. Molecules.

[111] van Geenen F.A.M.G., Franssen M.C.R., Miikkulainen V., Ritala M., Zuilhof H., Kostiainen R., Nielen M.W.F. (2019). TiO_2_ Photocatalyzed Oxidation of Drugs Studied by Laser Ablation Electrospray Ionization Mass Spectrometry. J. Am. Soc. Mass Spectrom..

[112] Koltsakidou A., Terzopoulou Z., Kyzas G., Bikiaris D., Lambropoulou D. (2019). Biobased Poly(ethylene furanoate) Polyester/TiO_2_ Supported Nanocomposites as Effective Photocatalysts for Anti-inflammatory/Analgesic Drugs. Molecules.

[113] Osathaphan K., Chucherdwatanasak B., Rachdawong P., Sharma V.K. (2008). Photocatalytic oxidation of cyanide in aqueous titanium dioxide suspensions: Effect of ethylenediaminetetraacetate. Sol. Energy.

[114] Ji Y., Zhou L., Ferronato C., Yang X., Salvador A., Zeng C., Chovelon J.-M. (2013). Photocatalytic degradation of atenolol in aqueous titanium dioxide suspensions: Kinetics, intermediates and degradation pathways. J. Photochem. Photobiol. A Chem..

[115] Wang Z., Srivastava V., Wang S., Sun H., Thangaraj S.K., Jänis J., Sillanpää M. (2020). UVC-assisted photocatalytic degradation of carbamazepine by Nd-doped Sb_2_O_3_/TiO_2_ photocatalyst. J. Colloid Interface Sci..

[116] Píšťková V., Tasbihi M., Vávrová M., Štangar U.L. (2015). Photocatalytic degradation of β-blockers by using immobilized titania/silica on glass slides. J. Photochem. Photobiol. A Chem..

[117] Khattak S.-R., Shaikh D., Ahmad I., Usmanghani K., Sheraz M.A., Ahmed S. (2013). Photodegradation and Stabilization of Betamethasone-17 Valerate in Aqueous/Organic Solvents and Topical Formulations. AAPS PharmSciTech.

[118] Ruokolainen M., Ollikainen E., Sikanen T., Kotiaho T., Kostiainen R. (2016). Oxidation of Tyrosine-Phosphopeptides by Titanium Dioxide Photocatalysis. J. Am. Chem. Soc..

